# A mouse model of cone photoreceptor function loss *(cpfl9)* with degeneration due to a mutation in *Gucy2e*

**DOI:** 10.3389/fnmol.2022.1080136

**Published:** 2023-01-09

**Authors:** Anna S. E. N. Naggert, Gayle B. Collin, Jieping Wang, Mark P. Krebs, Bo Chang

**Affiliations:** The Jackson Laboratory, Bar Harbor, ME, United States

**Keywords:** retinal membrane guanylate cyclase, leber congenital amaurosis, cone-rod dystrophy, mouse models, S opsin, M opsin

## Abstract

During routine screening of mouse strains and stocks by the Eye Mutant Resource at The Jackson Laboratory for genetic mouse models of human ocular disorders, we identified *cpfl9*, a mouse model with cone photoreceptor function loss*.* The mice exhibited an early-onset phenotype that was easily recognized by the absence of a cone-mediated b-wave electroretinography response and by a reduction in rod-mediated photoresponses at four weeks of age. By genetic mapping and high-throughput sequencing of a whole exome capture library of *cpfl9*, a homozygous 25 bp deletion within exon 11 of the *Gucy2e* gene was identified, which is predicted to result in a frame shift leading to premature termination. The corresponding protein in human, retinal guanylate cyclase 1 (GUCY2D), plays an important role in rod and cone photoreceptor cell function. Loss-of-function mutations in human *GUCY2D* cause LCA1, one of the most common forms of Leber congenital amaurosis, which results in blindness at birth or in early childhood. The early loss of cone and reduced rod photoreceptor cell function in the *cpfl9* mutant is accompanied by a later, progressive loss of cone and rod photoreceptor cells, which may be relevant to understanding disease pathology in a subset of LCA1 patients and in individuals with cone-rod dystrophy caused by recessive *GUCY2D* variants. *cpfl9* mice will be useful for studying the role of *Gucy2e* in the retina.

## 1. Introduction

The Eye Mutant Resource (EMR) has screened for and established robust genetic models of ocular diseases since 1988. Currently, retired mouse breeders from the production colony at The Jackson Laboratory (JAX) are screened by electroretinography (ERG), indirect ophthalmoscopy, and optical coherence tomography (OCT). Through the EMR screening program at JAX, the cone photoreceptor function loss 9 (*cpfl9*) mutant was discovered as displaying abnormal ERG responses. The mutation was determined to cause an autosomal recessive disease, mapping to Chromosome 11, and through whole-exome sequencing was identified as a 25 bp deletion in the *Gucy2e* gene.

GUCY2E (also known as GUCY2D in humans and rats, and alternatively referred to as GC1, RGC-1, RetGC1, and PGCE) is one of two retina-specific membrane guanylate cyclases that catalyze the synthesis of cGMP in the phototransduction cascade (Pugh et al., 1997; Baehr and Palczewski, 2007). cGMP, a second messenger, affects phototransduction, calcium homeostasis, neurotransmission, and vessel dilation (Kuhn, 2016; Tolone et al., 2019) and in the retina, altered levels have been associated with photoreceptor degeneration (Yang et al., 1999; Baehr et al., 2007).

In humans, over 100 reported pathogenic mutations in *GUCY2D* (ClinVar) have been associated with an array of retinal diseases. These include autosomal recessive Leber congenital amaurosis (LCA1; MIM#204000), autosomal dominant or recessive cone-rod dystrophy 6 (CORD6; MIM#601777), choroidal dystrophy, central areolar (CACD1; MIM#215500) and night blindness, congenital stationary, Type1I (CSNB1I; MIM#618555). In mice, 27 strains with mutations in *Gucy2e* are listed in the Mouse Genome Informatics database (Blake et al., 2009), but only a few studies with mice carrying the same targeted knockout allele have characterized the disease phenotype in detail (Yang et al., 1999; Coleman et al., 2004; Baehr et al., 2007). The *cpfl9* mutation was previously reported, in a brief meeting abstract and further characterization is provided here. Extinguished cone responses and an abnormal distribution of cone photopigments and GNAT2 are early phenotypes that are similar to those previously reported (Yang et al., 1999; Coleman et al., 2004; Baehr et al., 2007). However, unique observations of the *cpfl9* model include: (1) similar cone nuclei counts compared to wild-type (WT) control mice, but a reduced number of cone matrix sheaths at 12 weeks of age, (2) prominent differences in the cellular distribution of OPN1MW (M opsin) across the retina of mutants, and (3) a partial rod and cone photoreceptor cell loss by 9–12 months of age.

## 2. Materials and methods

### 2.1. Mice

The mice utilized in this study were bred and maintained in standardized conditions of the Production or Research Animal Facilities at The Jackson Laboratory (JAX, Bar Harbor, Maine, United States). Mice provided with an NIH31 6% fat chow diet and acidified water, were housed in a pathogen-free vivarium environment with a 14-h light/10-h dark cycle. All experiments were approved by the Institutional Animal Care and Use Committee (active ACUC Protocol #99089) and conducted in accordance with the ARVO Statement for the Use of Animals in Ophthalmic and Vision Research.

### 2.2. Origin of *cpfl9*

The *cpfl9* abnormality was discovered in a male knock-in mouse, B6;129P2-*Foxl2^tm1(GFP/cre/ERT2)Pzg^*/J (JAX stock #015854) through an ocular screen in the Eye Mutant Resource (EMR). The mouse presented with no cone ERG response at 5 weeks of age. Mice bearing the wild-type *Foxl2* allele were selected for breeding and phenotypic characterization to ensure that the ERG effects and associated disease phenotypes were not due to the *Foxl2* knock-in allele. The mutation was named *cpfl9* for cone photoreceptor function loss, further backcrossed to C57BL/6 (B6; JAX stock #000664) for two or three generations and has subsequently been maintained by repeated intercrossing to make a nearly fully inbred strain (Ne_4_F_19_), B6;129P2-*Gucy2e*^*cpfl9*^/Boc (JAX stock #033197). For simplicity, we retain the original mutation designation *cpfl9*, respectively, for the remainder of the paper, and unless otherwise indicated the genotype is homozygous for the mutant allele.

### 2.3. Gene mapping and identification

To determine the heritability and the chromosomal location of the *cpfl9* mutation, we mated homozygous *cpfl9* mice to DBA/2 J mice. The F_1_ mice, which did not exhibit retinal cone functional loss, were backcrossed to homozygous *cpfl9* mice to produce N_2_ mice. Tail DNA from N_2_ progeny was isolated as previously reported (Truett et al., 2000). A genome-wide scan using DNA pools from 12 affected (low cone amplitude) and 12 unaffected (normal cone amplitude) mice was carried out using 48 microsatellite markers evenly spaced throughout the genome. *cpfl9* co-segregated with markers on Chromosome 11. Subsequently, DNAs from 37 N_2_ offspring were genotyped individually using microsatellite markers to develop a refined structure map of the Chromosome 11 region encompassing the recessive mutation.

The causative mutation was discovered by comparing the whole exome sequences from a homozygous *cpfl9* mutant to reference *Mus musculus* sequences (GRCm38; Fairfield et al., 2011). There was a 25 bp deletion in exon 11 of the *Gucy2e* gene in the homozygous *cpfl9* mouse. To confirm the 25 bp deletion in *cpfl9*, we chose a pair of PCR primers (Forward: TTAGATCAGCTATGGACACC, Reverse: TAGTTCCAGCATGGCATAAG) to cover the 25 bp deletion for Sanger sequencing and genotyping. PCR was carried out using tail DNA from *cpfl9* homozygous, heterozygous and wild-type mice in a 20 μl PCR reaction containing 1 × PCR buffer (10 mM Tris–HCl pH 8.3, 50 mM KCl), 250 μM of dATP, dCTP, dGTP, and dTTP, 0.2 μM of the forward and the reverse primer, 1.5 mM MgCl_2_, and 0.6 U Taq polymerase. The following PCR program was used: 94°C for 90 s, followed by 35 cycles of 94°C for 30 s, 55°C for 45 s, and 72°C for 45 s with a final extension of 72°C for 2 min. For Sanger sequencing, PCR products were purified using HighPrep PCR magnetic beads (MagBIO, Gaithersburg, MD, United States, catalog #AC-60050). Sequencing reactions composed of 5 μl of purified PCR product and 1 μl of primer at 10 μmol/μl. Cycle sequencing of DNA samples was performed using Edge Biosystem’s BrilliantDye Terminator reaction kit Version 3.1. Sequencing reactions were purified using HighPrep DTR (MagBIO, catalog #DT-70050). Purified reactions were run on an Applied Biosystems 3730xl. Sequence data were analyzed using MacVector (v.18.2.5). For genotyping, PCR products were separated by electrophoresis on 3% MetaPhor (FMC, Rockland, ME, catalog #50184) agarose gels and visualized under UV light after staining with ethidium bromide. The sizes of the PCR products are 144 bp in wildtype and 119 bp in *cpfl9* mice.

### 2.4. Clinical evaluation and electroretinography

Eyes of mice used in linkage crosses and for characterization were dilated with 1% atropine ophthalmic drops (Bausch and Lomb Pharmaceuticals, Inc., Tampa, FL, United States) and evaluated by indirect ophthalmoscopy using a 78-diopter lens. Fundus photographs were taken with a Micron III *in vivo* bright-field retinal imaging microscope equipped with image-guided optical coherence tomography (OCT) capabilities (Phoenix Laboratories, Inc., Pleasanton, CA, United States).

For ERG evaluation of mutants, following an overnight dark adaptation, mice were anesthetized with an intraperitoneal injection of xylazine (80 mg/kg) and ketamine (16 mg/kg) in normal saline. Additional anesthetic was given if akinesia was inadequate. The equipment and protocol used here is as previously described (Chang et al., 2007). Briefly, dark-adapted, rod-mediated ERGs were recorded with responses to short-wavelength flashes over 4.0log units to the maximum intensity by the photopic stimulator. Cone-mediated ERGs were recorded with white flashes after 10 min of complete light adaptation. The signals were sampled at 1-ms intervals and averaged.

### 2.5. Histologic and immunohistochemical analysis

Mice were asphyxiated by carbon dioxide inhalation. Eyes were enucleated and immediately oriented to establish dorsal/ventral axes using a transilluminating dissecting microscope and marked at the nasal posterior artery using a tissue marking dye (Cancer Diagnostics Inc., catalog # 0762-S) prior to fixation. Tissue specimens were fixed overnight in cold methanol:glacial acetic acid:phosphate-buffered saline (3:1:4 by volume). Globes were paraffin-embedded and cut into 6-μm sections at the optic nerve and stained with hematoxylin and eosin. Stained eye sections were imaged on a NanoZoomer 2.0 digital scanner (Hamamatsu, Bridgewater, NJ). For immunohistochemical analysis, sections were deparaffinated in xylene, rehydrated through an alcohol gradient, and antigen retrieval was performed by microwave treatment in 10 mM citrate buffer. Sections were then blocked and permeabilized for 1 h at RT in blocking solution (2% normal donkey serum, 0.5% Triton X-100 in PBS), followed by primary antibody incubation at 4°C overnight. Fluorescent detection was performed with Alexa Fluor 488–conjugated goat anti-rabbit (#A21206) and Alexa Fluor 647-conjugated donkey anti-goat (#A32849TR) secondary antibodies (Invitrogen, Waltham, MA, United States). For detection of cone matrix sheaths, sections were co-stained with rhodamine-PNA (#RL-1072, Vector Laboratories, Burlingame, CA, United States) for 2 h at room temperature. For fresh cyrosections, enucleated eyes were oriented in a cryomold filled with optimal cutting temperature compound and snap-frozen in a 2-methyl butane in a lined metal box containing liquid nitrogen. Eyes were sectioned in 10-μm sections. Cryosections were fixed with 4% PFA in PBS for 10 min, incubated in blocking solution at RT for 30 min and incubated with primary antibody overnight. After washing in PBS, tissues were incubated in secondary antibodies/DAPI for 2 h at room temperature. After washing in PBS, samples were mounted in Vectashield and fluorescent signals visualized using a Zeiss Axio Observer with ApoTome 2.0 (Carl Zeiss, Germany).

Anti-guanyl cyclase E (GUCY2E; AB14788, Abcam), an antibody selected against the C-terminal end of murine retinal guanylyl cyclase 1 protein (AAC42081; according to the manufacturer), was tested on mutant and B6 control retinas. Other antibodies used were anti-rhodopsin (MAB5356; Millipore, Billerica, MA, United States), anti-blue opsin (OPN1SW; AB5407; Santa Cruz), anti-red/green opsin (OPN1MW; AB5405; Millipore, Billerica, MA, United States), anti-cone transducin (GNAT2; PA5-22340, Thermo Fisher), and anti-cone arrestin (ARR3, AB15282; Millipore, Billerica, MA, United States).

### 2.6. Retinal flatmounts

Following carbon dioxide inhalation, enucleated eyes were dissected mid-limbus in ice-cold 4% PFA. Eye cups were placed in 4% PFA for 2 h at 4°C, cut into petals, and retinas gently peeled from the retinal pigment epithelium (RPE)-choroid-sclera using a nitrocellulose membrane. After fixation, tissues were incubated in blocking solution for 2 h at RT followed by primary antibody for 3 days at 4°C with constant shaking. After washing in PBS, retinal flatmounts were immersed in a solution containing Alexa Fluor 488–conjugated goat anti-rabbit (#A21206) and Alexa Fluor 647-conjugated donkey anti-goat (#A32849TR) secondary antibodies (Invitrogen, Waltham, MA, United States). For cone staining, retinas were co-stained with rhodamine-PNA (#RL-1072, Vector Laboratories, Burlingame, CA, United States) for 2 days at 4°C with constant shaking. DAPI (#D1306, Invitrogen) was used as a nuclear stain. After several washes in PBS, tissues were post-fixed in 4% PFA for 10 min. Samples were rinsed through a glycerol gradient and then cover slipped in Vectashield mounting medium (H1000, Vector Laboratories). Samples were imaged at 10× using a Zeiss Axio Observer (Carl Zeiss). For cone counts, image stacks through the outer retina were acquired at dorsal and ventral regions using a Stellaris confocal microscope. To quantify cone matrix sheaths stained with PNA in these regions, the Find Maxima tool of Fiji was adjusted manually to optimize the identification of individual sheaths, with additional manual review of the image stack to identify sheaths that were not detected by the Find Maxima operation or to resolve overlapping sheaths where needed. For high-resolution imaging, image stacks were acquired on a Leica SP8 confocal microscope using a 63× glycerol objective and 1.5x digital zoom, 1.0 μm z-step. Image stacks were analyzed in Imaris 9.7.4 (Bitplane, South Windsor, CT, United States).

### 2.7. qRT-PCR analysis

Total RNA was isolated from postnatal day 18 (P18) retinas of B6 (*n* = 6) and homozygous *cpfl9* mice (*n* = 6) using TRIzol (Life Technologies)/chloroform treatment as previously described (Weatherly et al., 2022). RNA clean-up and DNase digestion was performed on an RNeasy spin column (Qiagen, Germantown, MD). Total RNA was eluted in RNase-free water and quantified using a Nanodrop ND1000 spectrophotometer (Thermo Fisher) and cDNA was generated using the Superscript IV First Strand Synthesis System (Thermo Fisher) according to manufacturer’s protocol.

Quantitative RT–PCR was performed in a 20 μl PCR reaction using the iTaq Universal SYBR Green SuperMix (Bio-Rad) and *Gucy2e*-specific primers positioned upstream (ex3F, CAGGGCTCAAGAACACCAGG; ex4R, GACCCCACAAAAGCCAGAGA) and downstream (ex12F, CTCAGCCACAGATGGAGGC; ex14R, CTTCGGATGCTGGAGCAGTA) of the *cpfl9* mutation and G*nat2*-specific primers (ex7F, TGTTTGATGTGGGAGGGCAG; ex8R, GATGGACGTAGCCGCAAAGA) on a CFX96 Real-Time PCR Detection System (Bio-Rad). The comparative CT method (ΔΔC_T_) was used to calculate a relative fold change in gene transcripts. Quantification was performed using 2*^−ΔΔCT^* with *Actb* (F, CCAGTTCGCCATGGATGACGATAT; R, GTCAGGATACCTCTCTTGCTCTG) as an internal control calibrator. Correct amplification of the target genes was verified by sequence and melting curve analyzes.

### 2.8. Western analysis

Enucleated eyes of four- and 12-week-old *cpfl9* and B6 mice were placed in ice-cold Dulbecco’s phosphate-buffered saline (Invitrogen) containing a proteinase inhibitor (EDTA-free; Roche, Indianapolis, IN, United States). Extraneous tissue adhering to the eye globe was trimmed away, and anterior segments were removed. The neural retina was separated from the RPE-choroid-sclera, and immediately frozen in liquid nitrogen. For retina lysate preparation, the retinal tissue from a single eye was gently homogenized using a motorized pestle (Thermo Fisher) in 80 μl ice-cold RIPA lysis buffer (0.5% sodium deoxycholate, 0.1% SDS, 1% NP-40, 1× PBS), supplemented with complete EDTA-free, proteinase inhibitor, Roche, Indianapolis, IN, United States. The retinal lysates were then centrifuged at 12,000×*g* for 5 min at 4°C, and the supernatants were collected. To determine protein concentration, a Bio-Rad Protein Assay (Bio-Rad) was performed according to manufacturer’s protocol and absorbance values recorded at 595 nm using a DU-530 spectrophotometer (Beckman Coulter, Indianapolis, IN). Equal amounts (20 μg) of lysate protein were fractionated by SDS-PAGE (10% Tris-glycine acrylamide gel) and transferred to a polyvinylidene difluoride membrane using standard protocols (Transblot Turbo system, Bio-Rad, Hercules, CA, United States). After blocking with 5% nonfat milk in TBST (137 mM sodium chloride, 20 mM Tris, and 0.05% Tween-20, pH 7.5), the blots were incubated with the indicated primary antibodies diluted in in TBST overnight and detected with either an anti-rabbit (#7074S, Cell Signaling) or anti-goat (#HAF017, R&D Systems) HRP-conjugated secondary antibody diluted 1:2000 in 5% nonfat milk (TBST). Signals were detected using Clarity Western ECL substrate (#1705060, Bio-Rad). Western blot bands were quantified by densitometry using Fiji (Schindelin et al., 2012) and the signals of the target protein were normalized to that of GAPDH. Images of immunoblots were acquired using the Syngene GeneFlash gel documentation system (Synoptics Ltd., Cambridge, United Kingdom) and immune-positive bands were digitally inverted in Fiji (Schindelin et al., 2012). Brightness and contrast were adjusted in Fiji.

Antibodies used for the studies included anti-arrestin (ARR3, 11100-2-AP, Proteintech, Rosemount, Illinois), anti-guanylate cyclase E (GUCY2E; AB14788, Abcam, Cambridge, UK), anti-cone transducin (GNAT2; PA5-22340; Thermo Fisher), anti-blue opsin (OPN1SW; AB5407; Santa Cruz), anti-red/green opsin (OPN1MW; AB5405; Millipore, Billerica, MA, United States) and anti-GAPDH (#2118, Cell Signaling, Danvers, MA).

### 2.9. Transmission electron microscopy

For ultrastructural analysis, enucleated eyes from six-week-old B6 and homozygous *cpfl9* mice were placed in 2.5% glutaraldehyde/2% paraformaldehyde fixative solution in phosphate buffer. Anterior segments were removed by incising along the limbus with Vannas scissors and discarded. The resulting eyecups were incubated in fixative for 2 h. The posterior segment was cut into four quadrants. Additional fixation with 0.25% glutaraldehyde/0.2% paraformaldehyde fixative was performed for 8 h followed by post-fixation with 1% osmium tetroxide. The dehydrated blocks were embedded in plastic. Posterior eyes were cut into ultra-thin sections, stained with uranyl acetate and lead citrate and examined with a JEM-1230 transmission electron microscope (JEOL, Ltd., Peabody, MA).

### 2.10. Variant analysis

Published missense and truncating alleles of the human *GUCY2D* gene were ascertained through UniProt (UniProt Consortium, 2021; Q02846) and ClinVar (Landrum et al., 2018), respectively.

### 2.11. Statistics

Results are represented as mean ± SEM except where noted. Statistical significance was determined in GraphPad Prism (Dotmatics, Boston, MA, United States) using unpaired two-tailed Student’s *t*-tests, two-way repeated measures ANOVA, and mixed effect ANOVA for data in which values were missing. Values of *p* < 0.05 were considered statistically significant.

## 3. Results

### 3.1. Discovery of cone photoreceptor function loss 9 (*cpfl9*) mice

The spontaneous mutation, *cpfl9* was discovered during an ERG screen in the Eye Mutant Resource (EMR) at The Jackson Laboratory. The proband, which presented with no cone ERG response at 5 weeks of age, was mated to a B6 female mouse with normal cone function. The resultant F_1_ progeny had normal cone ERG responses and were subsequently intercrossed. The intercrossed (F_2_) offspring (3 female and 4 male mice) were tested for cone function by ERG, and 29% (2/7) showed no cone ERG responses. These findings indicated that the *cpfl9* was an autosomal recessive mutation.

**Figure 1:**
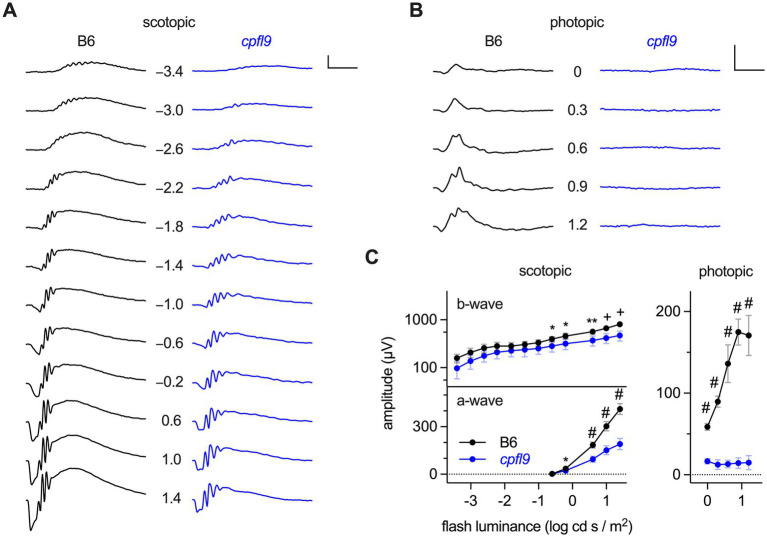
ERG responses of B6 and homozygous *cpfl9* mice at four weeks of age. **(A)** Representative scotopic ERG responses to light stimuli of increasing intensity as measured in a single eye of B6 (black traces) and homozygous *cpfl9* (blue traces) mice. Traces show the mean response recorded from two flashes. The flash illuminance in log cd s/m^2^ is indicated. **(B)** Photopic responses of the same eyes. Vertical scale bars in **A** and **B**, 200 μV; horizontal, 50 ms. **(C)** Light dose–response analysis showing scotopic b-wave and a-wave and photopic b-wave amplitudes of B6 (black circles, *n* = 7) and *cpfl9* (blue circles, *n* = 8). Values indicate mean ± SD. Two-way repeated-measures ANOVA indicated a statistically significant effect of strain on each response (scotopic b-wave, *F*(1, 13) = 16.5, *p* = 0.0013; scotopic a-wave, *F*(1, 13) = 131.6, *p* < 0.0001; photopic b-wave, *F*(1, 13) = 509.3, *p* < 0.0001). *Post hoc* multiple-comparison testing (Šídák) revealed statistically significant differences in response amplitude between strains as indicated by symbols (*, *p <* 0.05; **, *p* < 0.01; +, *p <* 0.001; #, *p* < 0.0001).

Detailed characterization of ERG responses indicated defects in both rod and cone photoreceptor cell function of homozygous *cpfl9* mice. B6 mice at 4 weeks of age produce robust ERG responses under scotopic and photopic conditions (Figures 1A,B). Scotopic responses of 4-week-old homozygous *cpfl9* mice, although reduced compared to B6 control mice, were still observable (Figure 1A). By contrast, photopic b-wave responses recorded in the presence of background light were undetectable in homozygous *cpfl9* mutants even at the highest stimulus intensities (Figure 1B). Analysis of response amplitudes as a function of light stimulus intensity indicated that the scotopic responses in homozygous *cpfl9* mice were roughly a third of those in B6 control mice (Figure 1C). Statistically significant differences in the scotopic a- and b-wave amplitudes of *cpfl9* and B6 mice were observed at the highest stimulus intensities and in the photopic b-wave amplitude at all intensities (Figure 1C). These results indicate that homozygous *cpfl9* mice have phototransduction pathway deficits.

### 3.2. Genetic and molecular basis of the *cpfl9* mutation

Pooled DNA for linkage analysis of *cpfl9* showed the mutation co-segregated with markers on Chromosome 11. Subsequently, genotyping of individual DNAs of 37 N_2_ offspring using microsatellite repeat markers was used to refine the critical region encompassing the mutation to a 9 cM interval between markers *D11Mit349* and *D11Mit4* (Supplementary Figure S1A).

**Figure 2:**
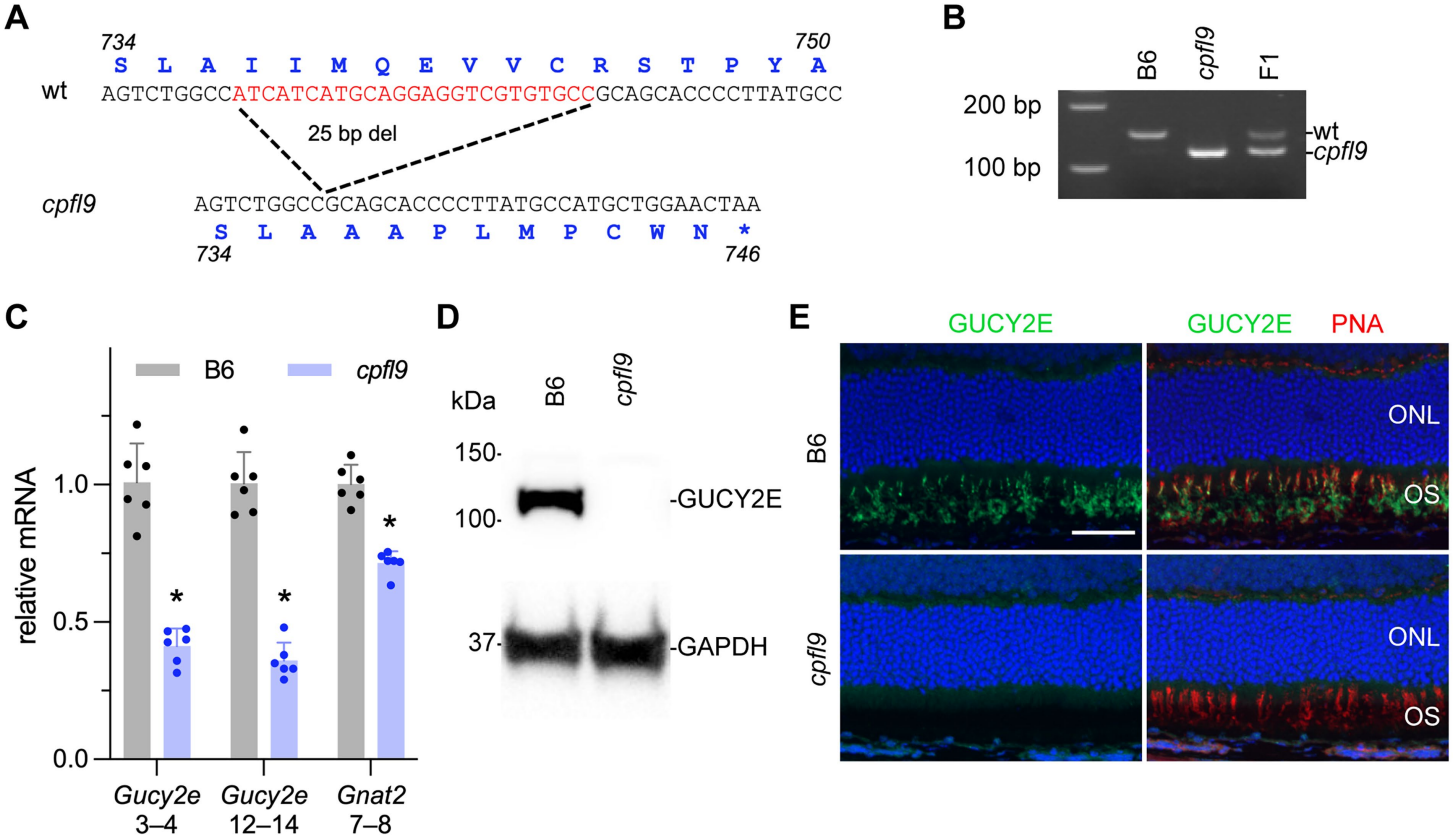
Molecular characterization of the *cpfl9* mutation. **(A)** Comparison of wild-type reference (Ensembl, ENSMUSG00000020890, GRCm39) and *cpfl9* genomic sequences of *Gucy2e* exon 11. A 25-bp deletion was observed in *cpfl9* mutant (shown in red). Corresponding amino acid sequences from *Gucy2e*-201 transcript are shown in blue. **(B)** Genotyping of homozygous *cpfl9*, B6 and F1 DNA by standard PCR/gel electrophoresis detects the WT allele at 144 bp and the mutant allele at 119 bp. F_1_ mice are heterozygous carrying one copy of each WT and mutant allele. **(C)** Quantitative RT-PCR of retinal lysates at P18 was performed using primers specific for *Gucy2e* (exons 3–4 and exons 12–14) and *Gnat2* (exons 7–8). The mean fold-change value ± standard error is shown. B6 (*n* = 6), gray; *cpfl9* (*n* = 6), blue; circles represent the values from individual mice. Asterisks indicate *p* < 0.0005, two-tailed Student’s t-test. **(D)** Representative lanes of an immunoblot with anti-GUCY2E in 4-week-old B6 and *cpfl9* retinal lysates. The predicted 120 kDa band was observed in B6 while absent in *cpfl9* retinas. The GAPDH loading control was observed at similar levels in both samples. Lysates from *n* = 5 *cpfl9* and *n* = 5 B6 mice were analyzed in separate lanes of the same blot. **(E)** Immunohistochemical staining with anti-GUCY2E (green) and PNA (red) shows absence of GUCY2E expression in photoreceptor OS of *cpfl9* retinas compared to B6. Images are representative of *n* = 5 *cpfl9* and *n* = 5 B6 mice. Scale bar, 50 μm.

To identify the causative mutation of *cpfl9*, whole exome sequences from a homozygous *cpfl9* mutant were compared with reference *Mus musculus* exome sequences (GRCm38). A 25 bp deletion in exon 11 (c.2209_2233del; Figure 2A) of the *Gucy2e* gene was discovered and subsequently confirmed to be specific to *cpfl9* by standard PCR and Sanger sequencing. PCR amplification of the region flanking the mutation revealed the expected 25 bp size difference between the mutant (119 bp) and wild-type (144 bp) alleles (Figure 2B). Chromatograms spanning the deletion are shown in Supplementary Figure S1B. This spontaneous deletion in exon 11 is predicted to result in a frame shift and premature termination at codon 746.

To determine whether the *cpfl9* deletion affects cone signaling early in phototransduction, we examined the expression of the gene encoding the alpha subunit of the cone-specific heterotrimeric G-protein, cone transducin (*Gnat2*) at postnatal day 18 (P18). Comparison of quantitative RT–PCR levels of *cpfl9* and B6 control mRNA revealed a significant reduction of *Gnat2* mRNA in *cpfl9* retinas compared to B6 retinas (relative normalized expression; two tailed Student’s t-test; *p* < 0.0001; Figure 2C).

To examine the effects of the 25 bp deletion on *Gucy2e* transcript levels, we performed quantitative RT-PCR on *cpfl9* and B6 control retinal lysates. We observed a significant reduction in *Gucy2e* transcripts upstream (exon 3–4) and downstream (exon 12–14) of the *cpfl9* deletion (Figure 2C; relative normalized expression; two tailed t-test; *p* < 0.00005 for both). The use of an antibody directed against a carboxy terminal peptide of GUCY2E did not detect GUCY2E in isolated *cpfl9* retinal lysates by western analysis (Figure 2D), or in the mutant photoreceptor outer segment (OS) by immunohistochemistry from 4-week-old mice (Figure 2E), indicating that *cpfl9* mutants fail to make a full length GUCY2E protein.

### 3.3. Age-dependent clinical, histological, and functional features of *cpfl9* mutants

Fundus and OCT images at 4 and 12 weeks of age (Supplementary Figure S2) did not differ between homozygous *cpfl9* mutants and B6 controls. However, at 9 months of age some mutants showed small discrete white spots that were not observed in control mice. Interestingly, in other mouse eye disease models (Kameya et al., 2002; Fogerty and Besharse, 2011; Kim et al., 2014; Rice et al., 2015; Zhu et al., 2019) these white spots have been found to correspond to activated immune cells (macrophages and/or microglia) in the subretinal space.

**Figure 3:**
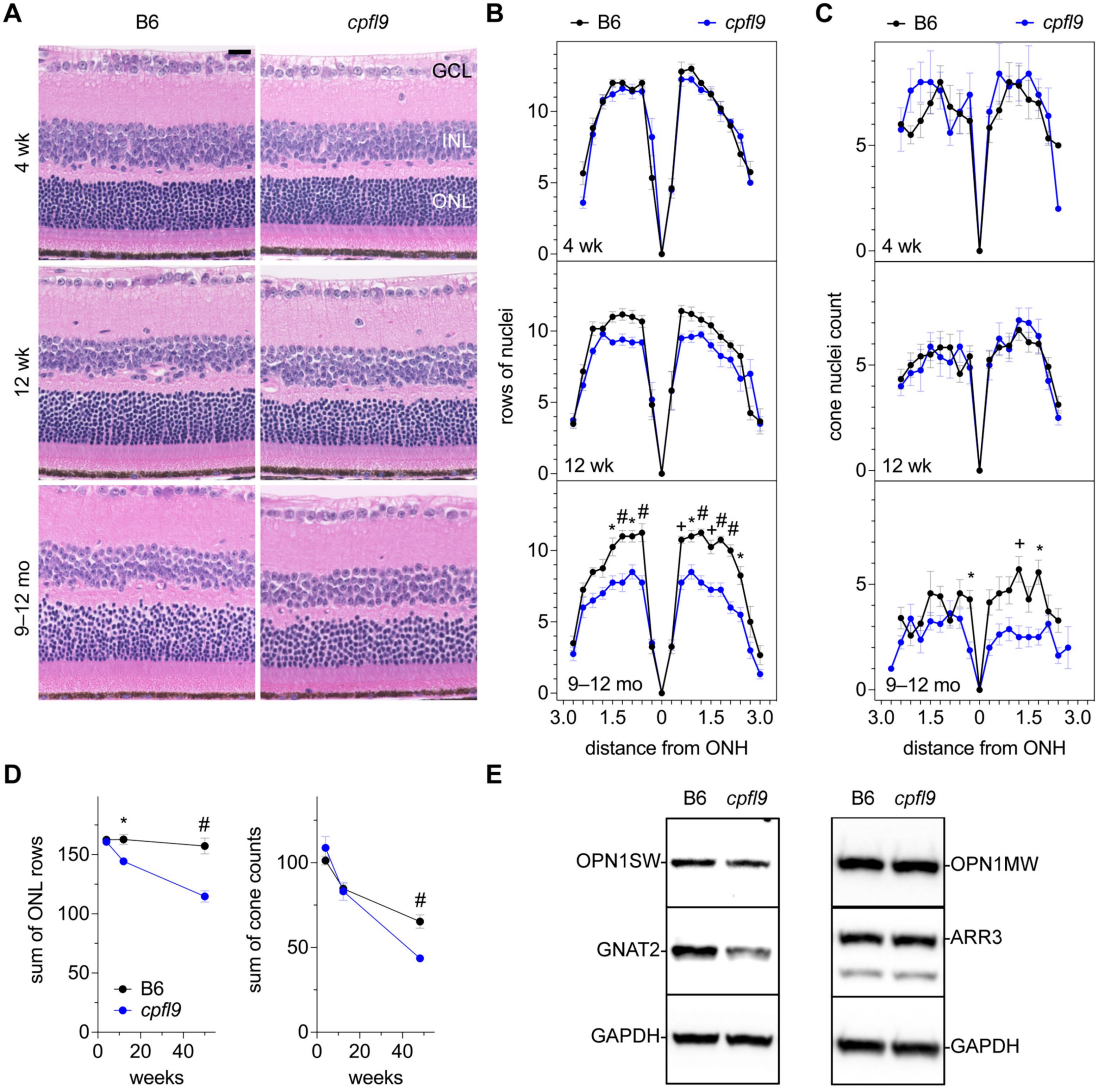
Effect of *cpfl9* mutation on retinal morphology and OS protein expression. **(A)** Retinal sections of *cpfl9* and B6 control eyes show similar hematoxylin-stained outer nuclear layer (ONL) thickness at 4 and 12 weeks of age. In contrast, at 9–12 months of age *cpfl9* mutants showed a thinned ONL compared to controls. GCL, ganglion cell layer; ONL, outer nuclear layer; INL, inner nuclear layer. Scale bar: 20 μm. Images are representative of slide scans from *n* = 4–8 *cpfl9* and *n* = 4–12 B6 mice at all ages. **(B)** Longitudinal quantitation of the number rows of ONL nuclei within a 50-μm length of retina as a function of distance from the optic nerve head (ONH). B6 control mice, black circles; *cpfl9* mice, blue circles. Values represent mean ± standard error of the mean (SEM); *n* = 4–6. Statistically significant differences from *post hoc* multiple comparison testing of row counts between the strains are indicated by symbols: *, *p* < 0.05; +, *p* < 0.001; #, *p* < 0.0001. **(C)** Longitudinal quantitation of cone photoreceptor nuclei within a 100-μm length of retina at regular intervals from the ONH. Values represent mean ± SEM; *n* = 4–12. Statistically significant differences from *post hoc* multiple comparison testing of cone counts between the strains are indicated by *, *p* < 0.05; +, *p* < 0.001. **(D)** Progressive change with age in the number of rows of ONL nuclei and cone nuclei counts summed over all retinal regions as measured in panels **B** and **C**. Values represent mean ± SEM; *n* = 4–12. *, *p* < 0.05; +, *p* < 0.001; #, *p* < 0.0001. **(E)** Western analysis of retinal lysates probed with antibodies against cone-specific OS proteins: S opsin (OPN1SW), M opsin (OPN1MW), cone arrestin (ARR3) and G protein subunit alpha transducin-2 (GNAT2). GAPDH was used as a loading control. Western analysis indicated decreased expression of GNAT2 in *cpfl9* compared to B6 retinas at 12 weeks of age. Images are representative of lysates from *n* = 4 *cpfl9* and *n* = 5 B6 mice analyzed in separate lanes of the same immunoblot.

To assess whether morphological changes to the retina occurred in homozygous *cpfl9* mice, representative histological sections were examined in mice at 4 and 12 weeks and at 9–12 months of age (Figure 3A). At 4 weeks of age the rows of ONL nuclei counted at intervals throughout the retina were comparable between *cpfl9* and B6 age- and sex-matched controls (Figure 3B). A decrease in row count was observed in *cpfl9* at 12 weeks of age, but a statistically significant effect of strain on row count was not observed. By 9–12 months of age, a statistically significant effect of strain was apparent (two-way mixed effect ANOVA, *F*(1,6) = 28.8, *p* = 0.0017). At this age, *post hoc* multiple comparison testing (Šídák) indicated a statistically significant loss of rows in the central retina of *cpfl9* compared to B6 mice (Figure 3B). The number of cone nuclei, identified based on nuclear morphology, was also assessed at intervals throughout the retina (Figure 3C). At 4 and 12 weeks of age, no effect of strain on cone nuclei counts was evident. However, at 9–12 months of age, a statistically significant effect of strain was apparent (two-way mixed effect ANOVA, *F*(1,13) = 27.6, *p* = 0.00016; B6, n = 7, *cpfl9*, *n* = 8). It should also be noted, that at 9–12 months of age the cone nuclei were morphologically abnormal and difficult to discern by this approach, even in B6 mice. As a further test of the loss of ONL nuclei, the rows of nuclei and cone counts at all locations were summed in samples for which the full retina was assessed (Figure 3D). A statistically significant decrease in rows was observed at 12 weeks of age (B6, 163 ± 9 rows; *cpfl9*, 144 ± 5 rows; *t*-test, *p* = 0.021; B6, *n* = 5, *cpfl9*, *n* = 3) and 9–12 months of age (B6, 157 ± 13 rows; *cpfl9*, 115 ± 10 rows, or 73% of the B6 value; Student’s *t*-test, *p* = 0.0020; B6, *n* = 4, *cpfl9*, *n* = 4; values represent mean ± SD). The sum of the cone count at all locations (Figure 3D) showed no significant difference at 4 and 12 weeks but a statistically significant decrease at 9–12 months of age (B6, 65 ± 10 cone nuclei; *cpfl9*, 44 ± 6 cone nuclei, or 68% of the B6 value; Student’s *t*-test, *p* = 0.00028; B6, *n* = 7, *cpfl9*, *n* = 8). Together, these results reveal a progressive degenerative loss of rod and cone photoreceptor cells with age in homozygous *cpfl9* mice.

To assess whether photoreceptor cell loss was accompanied by functional decline, we analyzed ERG response amplitudes in older mice. As photopic responses were extinguished at 4 weeks of age (Figure 1), the analysis was limited to scotopic responses. Statistically significant differences in both a- and b-wave scotopic response amplitudes of homozygous *cpfl9* and B6 mice were observed at 12 weeks and 9–12 months of age (Supplementary Figure S3A), and a progressive decrease in ERG response in homozygous *cpfl9* mice over this period was apparent (Supplementary Figure S3B). However, the ERG response of B6 mice also declined (Supplementary Figure S3B), despite a negligible change in ONL nuclei count (Figure 3D). A similar age-dependent decrease in the scotopic ERG response without photoreceptor loss has been reported in previous studies of B6 mice (Li et al., 2001; Ferdous et al., 2021). Thus, to determine whether ERG responses decline more rapidly in *cpfl9* mice, response amplitudes as a function of age were fit to a mono-exponential decay with lag (Supplementary Figure S3B), which has been used previously to model progressive photoreceptor degeneration in mice (Clarke et al., 2000; Collin et al., 2020). A statistically significant increase in the rate constant of the a-wave response amplitude decay curve was observed in homozygous *cpfl9* mice, indicating a more rapid loss of rod photoreceptor function than in B6 mice (Supplementary Figure S3B). This finding supports our histological evidence for progressive rod photoreceptor cell loss in homozygous *cpfl9* mice.

As indicated by ERG, the photopic b-wave amplitude was absent in homozygous *cpfl9* mice at 4 weeks of age (Figure 1). However, cone nuclei counting indicated the number of cone photoreceptor cells at 4 and 12 weeks of age was comparable in B6 and *cpfl9* mice (Figure 3D). To assess whether the loss of cone function was due to changes in the expression of cone proteins, we performed western analysis on retinal lysates from *cpfl9* mutant and B6 control mice at 12 weeks of age (Figure 3E). While similar levels of cone arrestin (ARR3) were detected in retinas from *cpfl9* and B6 mice, immunoblotting with anti-GNAT2 showed a 43% reduction of protein in *cpfl9* mutants compared to the B6 control (two-tailed Student’s t-test, *p* < 0.005). Moreover, protein levels of the cone opsins OPN1SW (blue or S opsin) and OPN1MW (green or M opsin) were decreased, while not significantly, in *cpfl9* compared to B6 retinas. These results indicate an effect of the *cpfl9* allele on expression of some but not all cone OS proteins.

### 3.4. Mislocalization of cone opsins and other OS proteins

**Figure 4:**
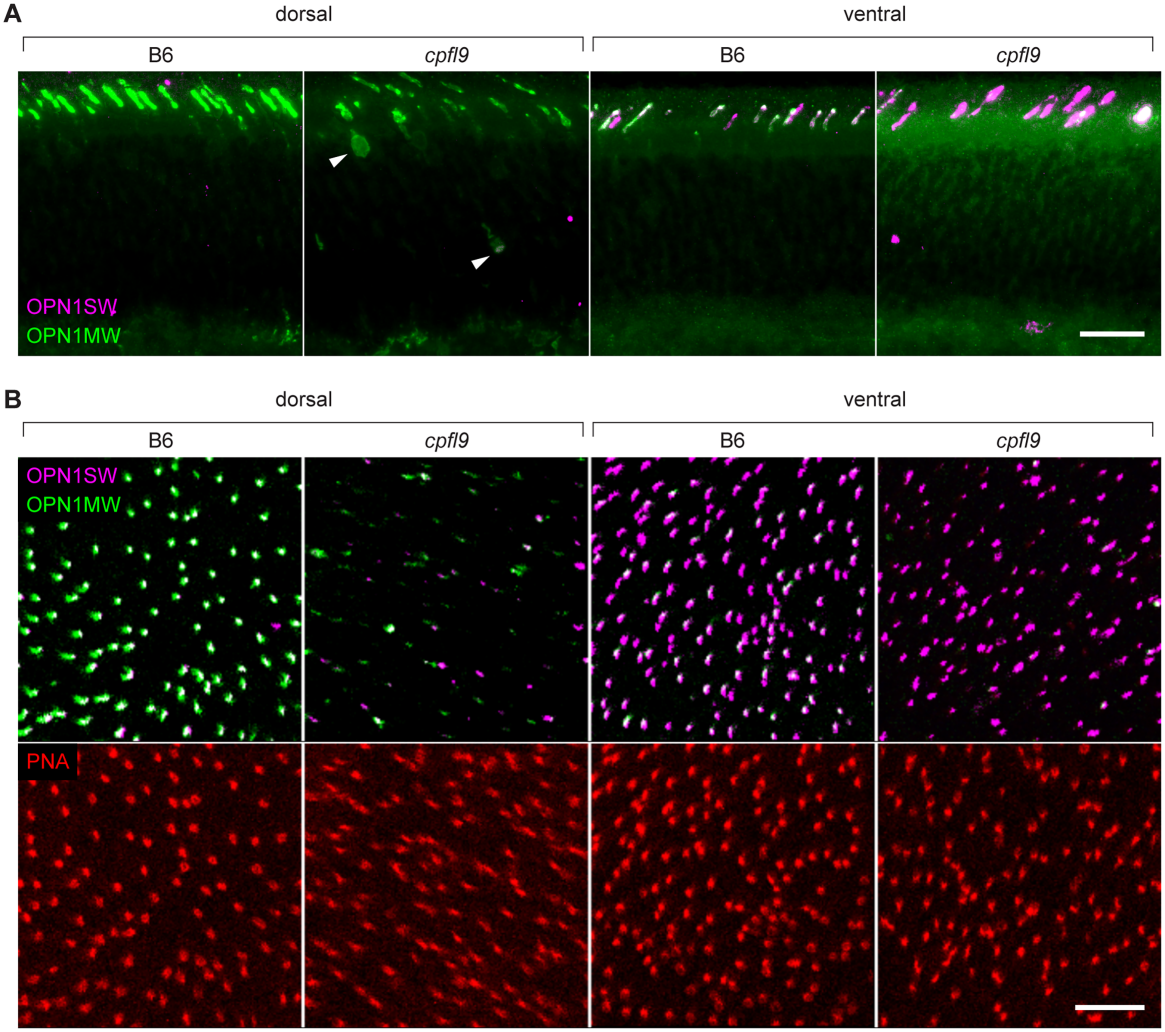
Immunostaining of cone opsins in *cpfl9* mutants and B6 controls at 12 weeks of age. **(A)** Thin retinal sections stained with anti-OPN1MW (green) and anti-OPN1SW (magenta). In the dorsal retina, where expression of M cones is predominant, mislocalization of OPN1MW to the cone cell soma (arrowheads) and pedicles was observed. In the ventral retina, there was a significant reduction of OPN1MW expression in dual-expressing cones. **(B)** Stellaris confocal imaging of retinal flatmounts. Scale bars in **A** and **B**, 10 μm. Images in **A** and **B** are representative of *n* = 4 *cpfl9* and *n* = 6 B6 mice.

To determine if the localization of cone opsin and other OS proteins was differentially affected by the *cpfl9* mutation, we stained dorsal/ventral-oriented sections with antibodies targeting OPN1SW, OPN1MW, and ARR3. At 12 weeks of age, while staining of rhodopsin was similar between mutant and controls (Supplementary Figure S4A), a pronounced mislocalization of cone opsins was observed in *cpfl9* mice (Figure 4A). In addition, staining of the OS protein, GNAT2 (Supplementary Figure S4B), was evident in the cone cell somata, pedicles, inner segment (ISs) and the distal OS of mutant retinas. ARR3 staining in the cone OS was similar between B6 and *cpfl9* mice at 4 weeks of age, however at 12 weeks, we detected intermittent regions of immune positivity in the distal OS adjacent to the RPE interface (Supplementary Figure S4C).

Examination of retinal flatmounts isolated from 12-week-old mice by confocal microscopy (Figure 4B) confirmed the immunohistochemical opsin results obtained from retinal sections. Approximate locations of areas used for quantifying cones are shown in Supplementary Figure S5. The expression of OPN1MW was decreased substantially in cone OSs of homozygous *cpfl9* mice compared to B6 mice, and the expression of OPN1SW in cone OSs of the ventral retina was also reduced, although to a lesser extent. Interestingly, although similar numbers of cone nuclei were observed between *cpfl9* mutants and B6 controls, PNA staining indicated a statistically significant reduction in the number of cone matrix sheaths in the ventral retina, as assessed by PNA staining (B6, 164 ± 15 cones, *n* = 4; *cpfl9*, 125 ± 6 cones, *n* = 4; unpaired two-tailed Student’s *t*-test, *p* = 0.008; counts were made in an area of 0.01 mm^2^; values represent mean ± SD). These results reveal a deficit in the accumulation of cone opsins in the OS of *cpfl9* mice and a loss of OS associated structures, indicating cone dystrophy.

### 3.5. Cone opsin mislocalization and cone dysmorphology

**Figure 5:**
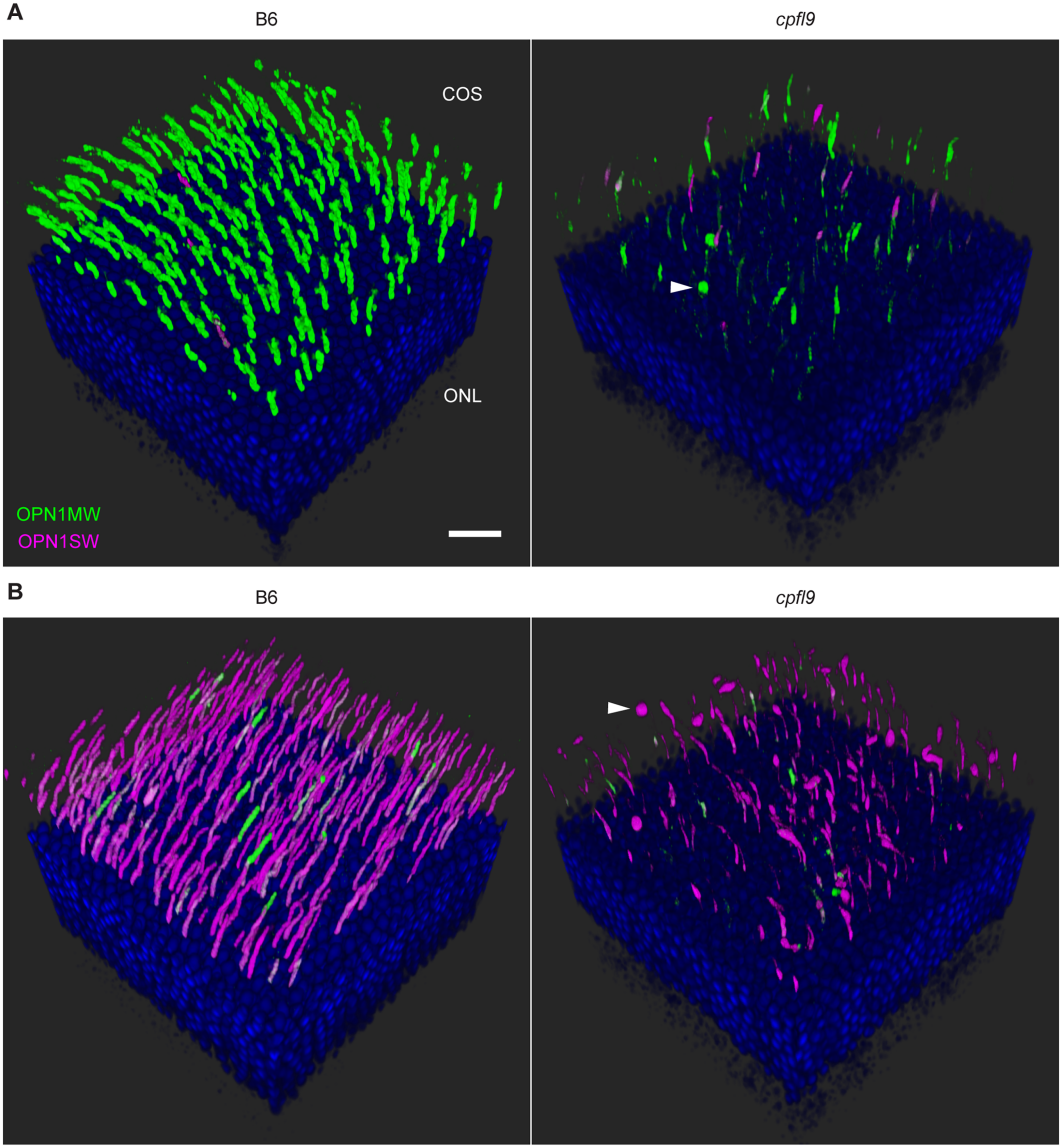
Confocal imaging of retinal flatmounts. B6 and *cpfl9* flatmount samples prepared at 12 weeks of age were stained with cone opsin antibodies and PNA as in Figure 4. **(A)** Dorsal retina. Cone OS (COS) were OPN1MW-positive (green) except for a few OS that were partly or completely OPN1SW-positive (magenta). OPN1MW staining was substantially reduced in the *cpfl9* retina. Round OPN1MW-positive objects were occasionally noted in the outermost retina (arrowhead). The ONL was revealed by DAPI staining (blue). Scale bar, 20 μm. **(B)** Ventral retina. OPN1SW-positive cones were observed in the ventral retina of B6 mice except for occasional cones that also were partly positive for OPN1MW. Reduced staining of both opsins was observed in the *cpfl9* ventral retina and round OPN1SW-positive objects were occasionally noted. Pseudocolors, symbols, and scale are the same as in panel **A**. Images are representative of *n* = 4 *cpfl9* and *n* = 5 B6 mice.

To understand the fate of cone opsins in the mutant mice, we further examined the stained retinal flatmounts by confocal microscopy at higher resolution (Figure 5). The OS as detected by cone opsin expression was uniformly shaped in both the dorsal (Figure 5A) and ventral (Figure 5B) regions of B6 retinas. By contrast, the accumulation of cone opsins in the OS of *cpfl9* mice was greatly diminished and the distribution of the protein within the OS was irregular. In some locations, opsins appeared to accumulate in round structures near the outer surface of the retina (Figures 5A, B, arrowheads). Thus, opsin is mislocalized, in part, to structures that are distinct from the OS and close to the subretinal space.

**Figure 6:**
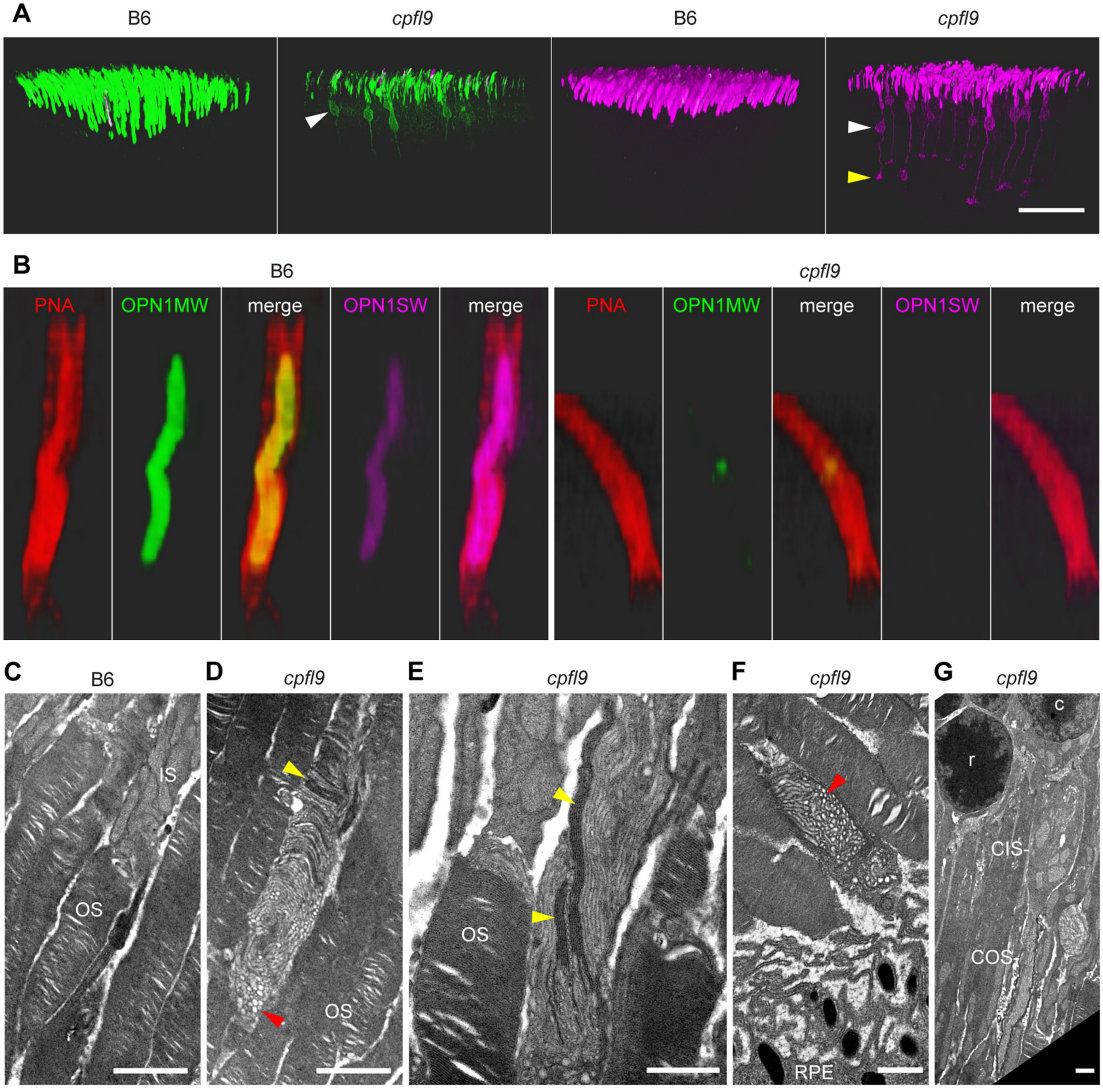
Opsin mislocalization and cone OS dysmorphology. **(A)** Confocal imaging of retinal flatmounts as described in Figure 5. In B6, OPN1MW (green) and OPN1SW (magenta) staining is observed only in the cone OS. By contrast, staining in *cpfl9* retinas is observed in cell bodies (white arrowheads) and structures toward the photoreceptor synapse. OPN1SW mislocalization reaches the cone pedicles (yellow arrowhead). Display settings were adjusted identically for all images in Imaris to emphasize opsin mislocalization. Scale bar, 50 μm. **(B)** Opsin deficit within the cone matrix sheath. A single cone OS from the stained B6 and *cpfl9* flatmounts was viewed in Imaris. In B6 cones, often both OPN1MW and OPN1SW staining were associated with PNA staining (red). By contrast, most cone sheaths in *cpfl9* flatmounts exhibited little cone opsin staining. **(C–G)** Ultrastructure of the outer retina in mice at 6 weeks of age by transmission electron microscopy. **(C)** Photoreceptor IS and OS in a B6 retina. Stacked disks were evident in the marked OS and in neighboring OSs. **(D)** An OS-like object in a homozygous *cpfl9* retina included disorganized membranes appearing as vesicles and tubules (red arrowhead). A few disks were stacked perpendicular to the OS axis as in B6 mice (yellow arrowhead). **(E)** Stacked membranes in OS-like objects of *cpfl9* mice were often much longer than in B6 mice and were oblique to the OS axis (yellow arrowheads). **(F)** OS-like objects exhibiting disorganized membranes (red arrowhead) were sometimes found in close proximity to RPE apical microvilli. **(G)** A *cpfl9* cone cell traced from its nucleus (c) included a disorganized OS containing oblique lamellar structures as in **E**. Images are representative of *n* = 4 *cpfl9* and *n* = 4 B6 controls. IS, inner segment; OS, outer segment; CIS, cone inner segment; COS, cone outer segment; RPE, retinal pigmented epithelium; c, cone cell nucleus; r, rod cell nucleus. Scale bars: C-G, 1 μm.

To assess the mislocalization of cone opsins further, we examined the retinal flatmount confocal data using Imaris display settings to visualize portions of the cone photoreceptor cells that were closer to the inner retina, including the cell body and synapse (Figure 6A). In B6 image datasets examined with these settings, a strong signal was observed in the OS region and no displacement of either OPN1MW or OPN1SW toward the inner retina was apparent. However, in *cpfl9* flatmount images, staining of these proteins was evident in the cell body (Figure 6A, white arrowheads) and in cellular processes directed toward the inner retina. OPN1SW staining appeared to include cone pedicles (Figure 6A). Thus, the cone opsins in homozygous *cpfl9* mice are also mislocalized to the cone cell bodies and pedicles.

As shown in Figure 4B, PNA staining was robust in homozygous *cpfl9* mice at 12 weeks of age, suggesting that cone matrix sheaths persist despite the decreased levels of cone opsins in the outer retinas of the mutant mice (compare *cpfl9* retinal flatmounts in 4B and 4C). To determine whether an alteration of cone sheath structure was correlated with the decreased opsin expression, we examined the high-resolution confocal datasets obtained from stained retinal flatmounts (Figure 6B). In both B6 and *cpfl9* retinas, cone sheaths as detected by PNA staining appear as elongated hollow structures in the outer retina. However, while OPN1MW and/or OPN1SW are present within the cone sheaths of B6 cone photoreceptors, these opsins are diminished in or absent from sheaths surrounding *cpfl9* cones. For example, only a small region of OPN1MW-positive staining is observed within the PNA envelope of the *cpfl9* cone shown in Figure 6B. These results suggest that cone sheaths are generated normally in *cpfl9* mice and are stable, while the contents of the associated OS changes with age.

Finally, to determine whether there were additional morphological defects in cone photoreceptors, we examined ultrathin retinal sections from homozygous *cpfl9* mutants (*n* = 4) and B6 controls (*n* = 4) at 6 weeks of age by transmission electron microscopy. The outer retina of B6 mice contained numerous OSs filled with disks in roughly perpendicular orientation to the OS axis (Figure 6C). In homozygous *cpfl9* mice, most OSs were similar to those in B6 mice. However, abnormal OS-like objects were also observed, which contained disorganized membranous structures appearing as vesicles and tubules, often adjacent to limited stacks of disks (Figures 6D–G). Long, electron dense lamellar stacks were sometimes observed to extended obliquely along the length of the OS-like objects (Figure 6E), which were found throughout the outer retina, including at the RPE (Figure 6F). Rarely, a disorganized OS was observed adjacent to an IS and/or soma of a cone cell (Figure 6G) suggesting that at least some OS-like objects derive from cone photoreceptors. The observation of disorganized vesicles and tubules in the OS-like objects of *cpfl9* mice is consistent with previous descriptions of *Gucy2e^tm1Gar^* targeted knockout mice (Baehr et al., 2007).

## 4. Discussion

The *cpfl9* mouse model described in the current work shares many of the features of a previously described null mutation in *Gucy2e* mice, which has been characterized in several studies (Yang et al., 1999; Coleman et al., 2004; Baehr et al., 2007). However, our observations, which include older mice than in earlier studies, provide additional insights into the progression of disease associated with *Gucy2e* mutation in mice and provide a closer examination of the morphological changes of the cones, as these appear to be the most significantly affected cell type.

### 4.1. GUCY2E structure

Guanylate (also guanylyl or guanyl) cyclases (GCs) are homo- and heterodimeric enzymes that catalyze the conversion of GTP to cGMP. In current models of rGC function, ligand binding is thought to result in tilting of the transmembrane segments and rotation of the guanylate cyclase domain relative to other protein domains, thereby positioning polypeptide regions from each monomer to form a competent enzymatic active site (Kuhn, 2016; Sharma et al., 2016; Maruyama, 2017). Enzyme activation is also influenced by rGC binding partners in the cytosol, which interact with intracellular domains to regulate formation of the active site (Kuhn, 2016; Sharma et al., 2016; Maruyama, 2017).

**Figure 7:**
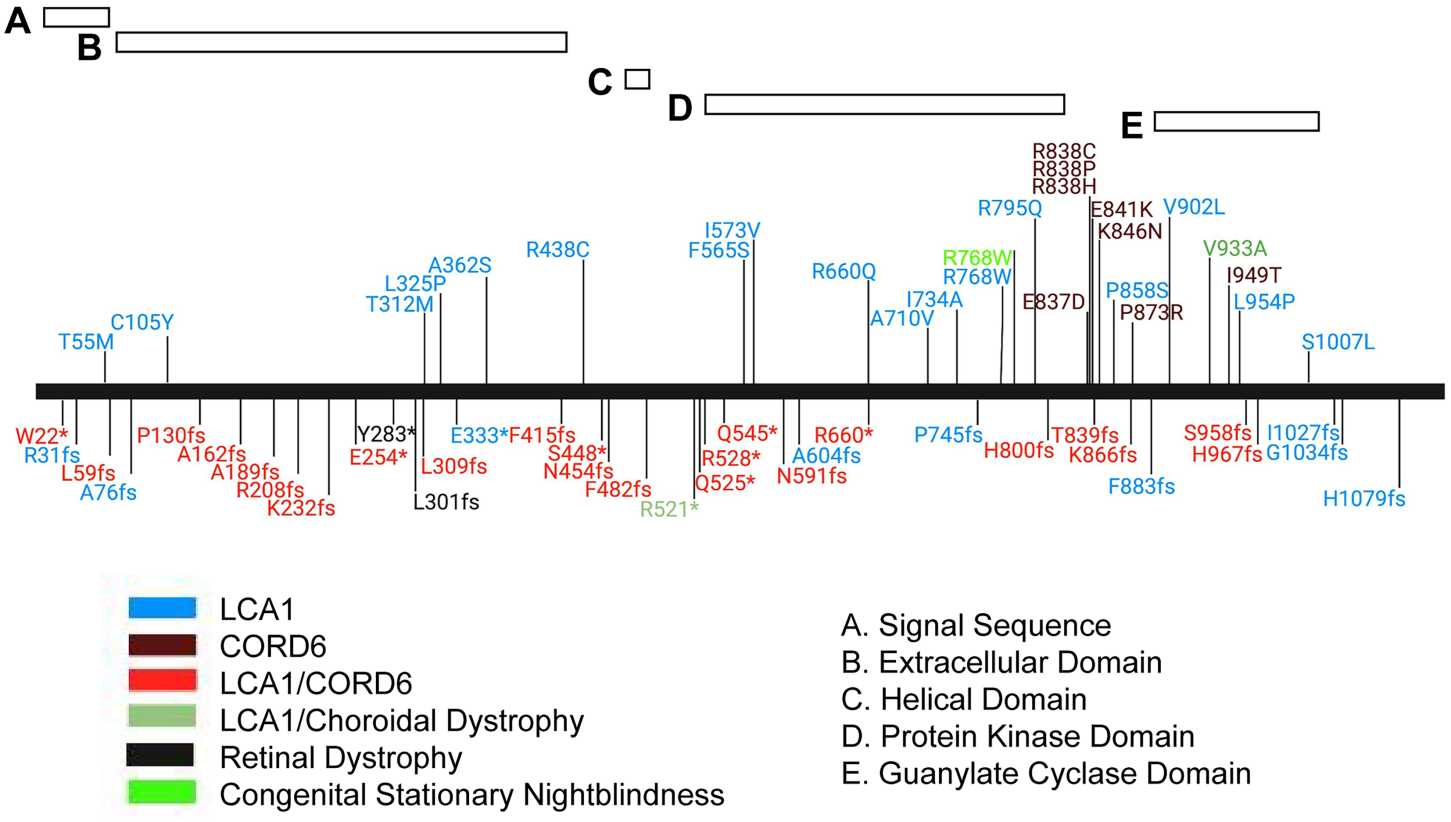
Mutation spectrum of the human GUCY2D protein. Schematic representation of the GUCY2D protein with relative position of protein domains. Alleles are localized by vertical lines and named. *, translation termination; fs, frameshift. Phenotype annotations are color coded in the allele symbol. LCA1, Leber congenital amaurosis 1, CORD6, cone-rod dystrophy 6.

Retinal guanylate cyclase 1 (GUCY2E; GUCY2D in humans) is a homodimeric enzyme belonging to the subfamily of receptor guanylate (also guanylyl or guanyl) cyclases (rGCs). These proteins localize to cellular membranes and possess extracellular, transmembrane, and intracellular domains that regulate the enzymatic activity of the guanylate cyclase domain (Maruyama, 2017). The structure of GUCY2E includes a processed N-terminal signal sequence and extracellular domain of unknown function and unknown ligand-binding capabilities, a single helical transmembrane domain, a kinase homology or pseudokinase domain, a linker segment of roughly 50 amino acids, and finally the guanylate cyclase catalytic domain (Figure 7). GUCY2E accumulates in the OS of rod and cone photoreceptor cells, where it plays an essential role in restoring cGMP levels in the dark following light excitation (Boye, 2014). A related protein, GUCY2F is unique to rod photoreceptor cells and contributes to cGMP production. In rods, rhodopsin excitation by light results in cGMP depletion by activating the heteromeric G protein transducin and consequently cGMP phosphodiesterase; decreased cGMP leads to closing of cGMP-gated cation channels. Recovery occurs in response to the ensuing decrease in intracellular calcium, as low calcium stimulates guanylate cyclase activating proteins (GCAPs) 1 and 2 to activate GUCY2E and GUCY2F. A similar recovery process occurs in cone photoreceptors in response to light excitation of cone pigments, activation of cone transducin and phosphodiesterase, and requires GUCY2E activation to restore cGMP and intracellular calcium levels (Karan et al., 2010).

In addition to its role in phototransduction, GUCY2E interacts with proteins of the phototransduction cascade and is implicated in the directional transport of some of these proteins to the OS, such as phosphodiesterase subunits and guanylate cyclase activating proteins (Karan et al., 2008, 2010). Additionally, GUCY2E has been shown to be modulated by bicarbonate (Duda et al., 2015, 2018; Makino et al., 2019), which may influence its activity in phototransduction and/or directional protein transport. Other roles for GUCY2E may yet be identified, by analogy with other rGCs that are activated or regulated by extracellular ligands, such as natriuretic factor, thrombin, guanylin and uroguanylin, or by the binding of calcium or chloride ion (Sharma et al., 2016; Maruyama, 2017).

Mutations in GUCY2D that lead to cone-rod dystrophy (CORD6; MIM#601777), choroidal dystrophy, central areolar (MIM#215500) and night blindness, congenital stationary, Type1I (CSNB1I; MIM#618555) are primarily found in the carboxyl terminal, dimer portion of the gene. However, mutations that lead to Leber congenital amaurosis 1 (LCA1; MIM#204000) are found throughout the gene, including the region encoding the carboxy terminus of the protein (Figure 7). The mutation spectrum of human GUCY2D does not reveal a strong genotype–phenotype correlation, although most pathogenic missense mutations are found in the kinase homology and guanylate cyclase catalytic domains (Figure 7). A cluster of missense mutations leading to autosomal dominant cone-rod dystrophy is found in a helical region between the kinase homology and cyclase domains. Truncating mutations are found throughout the protein sequence. Interestingly, most of them are annotated in ClinVar as causing both LCA1 and CORD6. This may indicate the presence of modifier genes in the human population that affect disease onset and severity.

### 4.2. Phototransduction defects in *cpfl9* mice

Our functional analysis of the *cpfl9* model at 4 weeks of age revealed that the photopic ERG response was abolished and the scotopic ERG response was significantly reduced compared to that of control mice. However, at this age, retinal histology was normal and counts of ONL and cone nuclei did not reveal a statistically significant reduction in the number of photoreceptor cells compared to controls. Together, these findings suggest that the diminished ERG responses in young *cpfl9* mice are due to deficits in the cone and rod phototransduction pathways rather than a defect in photoreceptor development or rapid photoreceptor cell degeneration. In previous work, null mutations in the guanylate cyclase gene in the GUCY1*B chicken were found to decrease retinal cGMP production by 5- to 10-fold (Semple-Rowland et al., 1998) and abolish ERG responses in both rod and cone photoreceptor cells (Ulshafer et al., 1984). In mice, *Gucy2e* knockout abolished cone ERG responses; however, the presence GUCY2F in rod photoreceptors apparently allows a detectable but decreased rod ERG response (Yang et al., 1999; Baehr et al., 2007). A similar effect may explain the decreased rod response in young *cpfl9* mice.

### 4.3. Photoreceptor cell loss in *cpfl9* mice

The *cpfl9* model appears to result in a milder cone degenerative disease than other animal models of *GUCY2D* deficiency. In the GUCY1*B chicken, retinal morphology is normal at day 1 post-hatch, but loss of photoreceptor cell nuclei is observed starting at day 21 post-hatch and is complete by ∼3.5 months of age (Ulshafer et al., 1984). In this mutant, in-frame replacement of exons 4–7 with an 81 bp segment homologous to a portion of exon 9 leads to loss of the protein as assessed with antibodies directed to the intracellular domain (Semple-Rowland et al., 1998). Thus, the mutation is considered a null allele, which may explain the severe degenerative phenotype. In the *Gucy2e* knockout mouse, initial characterization indicated that cone cell nuclei were rapidly lost by five weeks of age (Yang et al., 1999). However, another study of mice bearing the same knockout allele indicated that cones survived for considerably longer periods, requiring six months for complete loss in the inferior retina and longer for the superior retina (Coleman et al., 2004). In the knockout allele, exon 5 is replaced with a *neo* cassette, resulting in premature termination of the protein. No polypeptide was detected in the knockout mice using a C-terminal antibody, indicating that the mutation was a null allele. By contrast, in *cpfl9* mice, cone cell nuclei numbers do not differ significantly from those in B6 control mice at 4 and 12 weeks of age. Even at 9–12 months of age, the sum of cone nuclei counts obtained at intervals throughout the *cpfl9* retina was 68% of that in B6. Thus, cone cell loss in *cpfl9* mice appears to progress less rapidly than in the GUCY1*B chicken and the *Gucy2e* knockout mouse.

The *cpfl9* model also exhibits rod cell loss, but its progression is intermediate between that of other animal models. In the GUCY1*B chicken, rod cell loss was first detected at about the same age as cone cell loss and was complete by 6–8 months of age (Ulshafer et al., 1984). In *Gucy2e* knockout mice, however, no rod cell loss was reported among the oldest mice examined at 6 months of age (Yang et al., 1999; Coleman et al., 2004). Our analysis of *cpfl9* mice revealed a small but statistically significant decrease in the number of rod cell nuclei across the retina at 12 weeks of age, and a further decrease at 9–12 months of age, compared to controls. This decline was accompanied by a decrease in rod cell function as measured by ERG. Thus, *cpfl9* mice model *GUCY2D-*associated cone-rod dystrophy. Interestingly, the difference in rod cell degeneration of *cpfl9* mutant and *Gucy2e* knockout mice parallels observations of LCA1 patients, in which abnormal cone function was accompanied by a range of rod dysfunction from near normal (<0.5 log_10_ unit rod loss) to severe (>3.5 log_10_ units rod loss) as determined by full-field sensitivity testing (Jacobson et al., 2017). Similarly, two studies that assessed nyctalopia among LCA1 patients, an indication of rod dysfunction, revealed that most patients were normal, with 14 and 38% reporting the condition (Bouzia et al., 2020; Hahn et al., 2022). These findings establish that the severity of rod dysfunction in LCA1 patients is highly variable, possibly due to *GUCY2D* allelic differences (Sharon et al., 2018; Bouzia et al., 2020) and/or interactions with background modifier gene variants (Jacobson et al., 2022). Comparison of a series of *Gucy2e* alleles, possibly including the knockout and *cpfl9* alleles, in mice of the same genetic background may be informative for understanding the molecular basis of such variation.

At the molecular level, our analysis of *cpfl9* genomic DNA revealed a homozygous 25 bp deletion in the *Gucy2e* gene, significant downregulation of *Gucy2e* mRNA in *cpfl9* mice compared to B6 controls, and undetectable full-length protein in western analysis using a carboxy-terminal antibody against GUCY2E. Residual expression of *Gucy2e* in *cpfl9* mutants suggests partial escape of nonsense mediated decay (Supek et al., 2021), perhaps due to the presence of alternative splice isoforms or as a consequence of aberrant splicing resulting from the 25 bp deletion. However, since *Gucy2e* retinal transcripts were detected in *cpfl9* mutants, we cannot exclude the possibility that the 25 bp deletion results in a truncated form of the protein that retains partial GUCY2E function as conferred by the extracellular, transmembrane, and/or kinase homology domains. The truncated protein may lead to differences in the cone and rod degenerative phenotypes as compared to *Gucy2e* knockout mice, which may fail to express any protein, as described above. Further characterization of *Gucy2e* transcripts and/or amino-terminal GUCY2E polypeptides in the *cpfl9* mutant will be necessary to determine if the mutation results in complete loss of the GUCY2E protein.

### 4.4. Effect of the *cpfl9* allele on cone OS protein distribution

At 12 weeks of age, cone cell bodies of homozygous *cpfl9* seem to remain intact and there appears to be little effect of the *cpfl9* allele on cone opsin levels in retinal lysates. Nevertheless, OPN1MW and OPN1SW staining is greatly diminished in the cone OS and the proteins are mislocalized, both to objects near the outer retina and to the cone photoreceptor cell soma and synapse. The OPN1MW and OPN1SW objects detected by immunohistology in the outer retina may correspond to the abnormal membraneous particles containing intracellular vesicles, which were detected by TEM imaging and were found throughout the OS, extending as far as the subretinal space. Mislocalization of two other cone OS proteins, GNAT2 and ARR3, was also observed in *cpfl9* mice. The mislocalization of OS proteins in *cpfl9* mutants appears to be the result of an early defect of cone photopigments that are differentially expressed across the retina. The distribution of photopigments in cones that express OPN1MW is more profoundly affected; OPN1MW is observed in the cell soma and cone pedicles in the dorsal retina while a reduced expression is found in the ventral retina where there is a large population of cones expressing both pigments. These results strengthen similarities to the *Gucy2e* knockout mouse (Coleman et al., 2004; Coleman and Semple-Rowland, 2005; Baehr et al., 2007) and provide support for a model in which GUCY2E functions in the trafficking of membrane vesicles containing transmembrane and peripheral OS proteins from the endoplasmic reticulum and Golgi apparatus to the base of the photoreceptor cilium (Karan et al., 2010).

It is also possible that OS protein mislocalization in *cpfl9* mice results from defects in processes other than protein trafficking to the cilium. OS development and maintenance requires protein and lipid transport across the photoreceptor connecting cilium, followed by the formation and stacking of OS disks (Goldberg et al., 2016; Barnes et al., 2021). Disruption of the OS is expected to compromise the sequestration of OS proteins, which may subsequently become mislocalized to the IS, cell body, and synapse. In support of a defect in OS development and maintenance, ultrastructural analysis of *cpfl9* mice revealed cone OS defects, including the presence of overgrown, misaligned lamellae, accumulation of disorganized membrane vesicles and tubules, and displaced OS-like objects in the subretinal space. Ultrastructural defects in rod OSs were not detected; these may be rare due to the presence of functional GUCY2F in rods. A similar ultrastructural phenotype of misaligned OS disks combined with mislocalized OS proteins and progressive photoreceptor cell loss has been observed in several mouse retinal dystrophy models bearing mutations in genes involved in OS development and maintenance, including *Cngb1-Garp1-Garp2* (Zhang et al., 2009), *Kif3a* (Avasthi et al., 2009), *Prom1* (Zacchigna et al., 2009; Dellett et al., 2014), and *Rp1* (Gao et al., 2002; Liu et al., 2003; Song et al., 2014). As recently summarized (Goldberg et al., 2016), the proteins encoded by these genes are implicated in cargo transport across the connecting cilium (KIF3A), disk formation (CNGB1-GARP1-GARP2, PROM1, RP1), disk stacking (RP1), and disk stabilization (CNGB1-GARP1-GARP2). Of note, *Cngb3* knockout mice, which specifically target a homolog of *Cngb1* in cone photoreceptors, exhibit a similar combined phenotype (Carvalho et al., 2011; Xu et al., 2011), including defects in cone OS ultrastructure that are strikingly similar to those of *cpfl9* and *Gucy2e* knockout mice (Baehr et al., 2007). Thus, given the phenotypic similarities among these retinal dystrophy models and *Gucy2e* mutant mice, it is possible that GUCY2E functions in ciliary transport or disk formation, stacking and/or stabilization (Karan et al., 2008).

### 4.5. Cone matrix sheaths in the absence of cone opsins

At 12 weeks of age, cone matrix sheaths were abundant in *cpfl9* mice. However, based on our confocal analysis, the opsin content within the sheaths was low, and often no OPN1MW or OPN1SW staining was detected within the PNA-stained envelope. The persistence of cone sheaths over several months despite the absence of a cone opsin has been observed in *Opn1mw* knockout mice (Deng et al., 2018, 2019), although ultimately sheath loss is observed by PNA staining and is restored by delivery of a functional *Opn1mw* gene (Deng et al., 2019). These results, together with our findings, lead to the question, how do cone sheaths form when OPN1MW is absent, as in *Opn1mw* knockout mice, or mislocalized, as in *cpfl9* mice? A possible explanation relates to the developmental switch in cone OS opsin expression observed in mice and other vertebrates (Röhlich et al., 1994; Szél et al., 1994; Savelli et al., 2018). At early developmental stages, only short wavelength opsin accumulates in the OS, while at later stages both short and long wavelength opsins are present, often depending on topographic position within the retina (Szél et al., 1992; Applebury et al., 2000; Eldred et al., 2020). Mice express OPN1SW at early postnatal developmental stages but coexpress both OPN1SW and OPN1MW in a dorsal-ventral concentration gradient at later stages. For example, OPN1SW opsin but not OPN1MW opsin was observed in the OS of mouse cones at P7, while coexpression of both proteins were observed in cones at P14 (Glaschke et al., 2010). Thus, in *Opn1mw* knockout and *cpfl9* mice it is possible that cone sheaths form in early development as OPN1SW is expressed and remain stable for months thereafter, despite deficits in OPN1MW synthesis or OS maintenance. This proposed mechanism raises the possibility that GUCY2E is not needed for early OS development and sheath formation in OPN1SW cones but is required as part of the subsequent developmental switch from exclusive OPN1SW expression to combined OPN1MW coexpression. Examination of cone opsin expression in *cpfl9* mice at early developmental stages may help to address this possibility.

### 4.6. Summary

This work provides a detailed characterization of cone dysmorphology and rod and cone photoreceptor loss in *cpfl9* mice, which carry a novel mutant allele of *Gucy2e.* The mild cone-rod dystrophy disease phenotype exhibited by this mouse model may make it attractive for testing therapeutic interventions designed to mitigate the human retinal diseases caused by *GUCY2D* mutations.

## Data availability statement

The original contributions presented in the study are included in the article/Supplementary material, further inquiries can be directed to the corresponding author.

## Ethics statement

The animal study was reviewed and approved by The Jackson Laboratory Institutional Animal Care and Use Committee.

## Author contributions

GC, MK, and BC: conception and experimental design. AN, GC, JW, MK, and BC: execution of experiments. AN, GC, MK, and BC preparation of figures, manuscript. MK and BC: resources. All authors contributed to the article and approved the submitted version.

## Funding

Research in this publication was supported by the National Eye Institute of the National Institutes of Health under award numbers R01EY019943 (to B.C.) and R01EY027305 (to Patsy M. Nishina and M.P.K). The authors also wish to acknowledge the National Cancer Institute of the National Institutes of Health under award number P30CA034196 for support of core services.

## Conflict of interest

The authors declare that the research was conducted in the absence of any commercial or financial relationships that could be construed as a potential conflict of interest.

## Publisher’s note

All claims expressed in this article are solely those of the authors and do not necessarily represent those of their affiliated organizations, or those of the publisher, the editors and the reviewers. Any product that may be evaluated in this article, or claim that may be made by its manufacturer, is not guaranteed or endorsed by the publisher.
