# Supplementary material for: A mouse model of cone photoreceptor function loss (cpfl9) with degeneration due to a mutation in Gucy2e
Source: Front Mol Neurosci. 2023 Jan 9;15:1080136. doi: 10.3389/fnmol.2022.1080136 (PMC9868315; doi:10.3389/fnmol.2022.1080136)
Supplement: Supplementary file 1 [file Image_1.PDF]

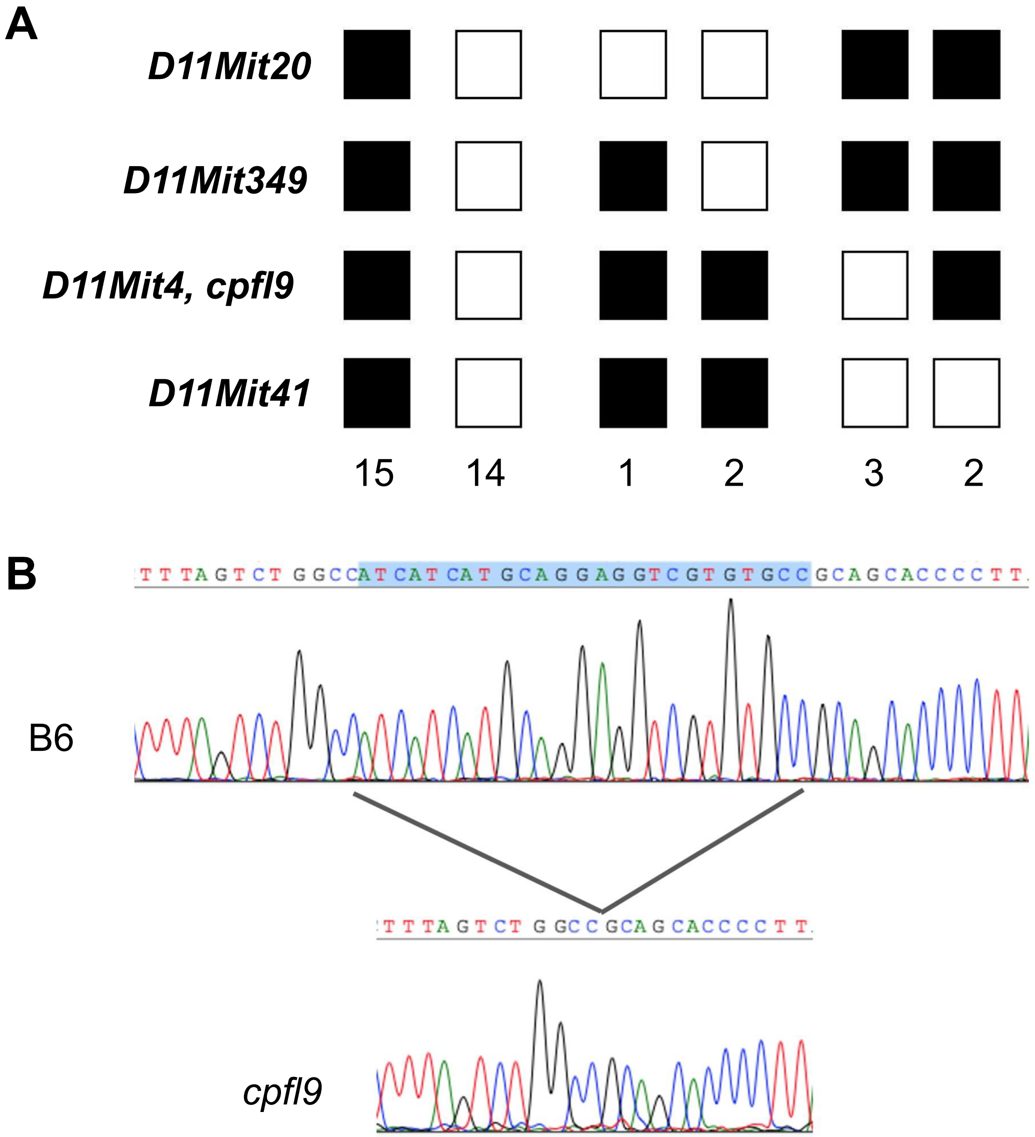

Figure S1. (**A**) Recombination mapping of *cpfl9* to mouse chromosome 11. Genetic analysis indicates *cpfl9* is caused by an autosomal recessive mutation that maps to mouse Chromosome 11 between D11Mit349 and D11Mit4. (**B**) Comparison of wild-type reference genomic sequence (ENSMUSG00000020890, GRCm39) and *cpfl9* genomic sequence of *Gucy2e* exon 11. A 25 bp deletion was observed in *cpfl9* mutant is shown in red. Corresponding amino acid sequences are shown in blue.
