# Supplementary material for: A mouse model of cone photoreceptor function loss (cpfl9) with degeneration due to a mutation in Gucy2e
Source: Front Mol Neurosci. 2023 Jan 9;15:1080136. doi: 10.3389/fnmol.2022.1080136 (PMC9868315; doi:10.3389/fnmol.2022.1080136)
Supplement: Supplementary file 2 [file Image_2.PDF]

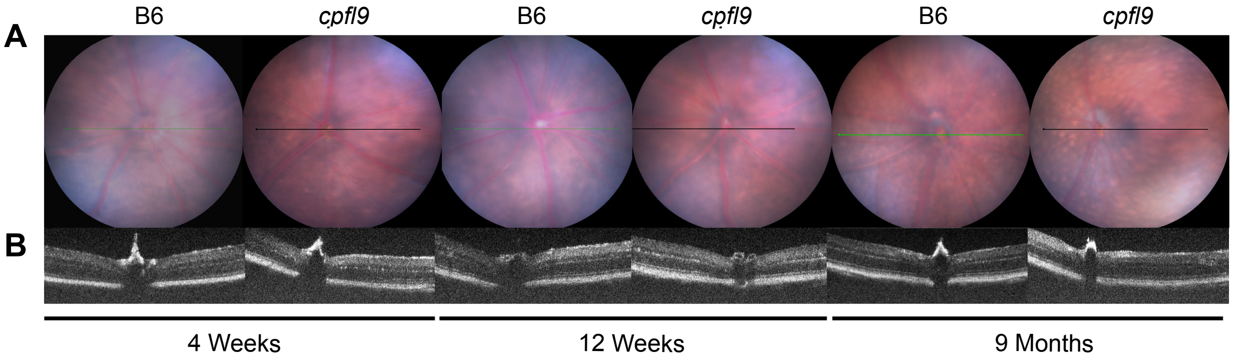

Figure S2. (**A**) Fundus and (**B**) corresponding OCT images of *cpfl9* mutants and controls at 4 weeks, 12 weeks and 9 months of age revealed fundus spots in some mutants but not control mice of comparable ages. Images are representative of n = 7 *cpfl9* and n = 10 B6 mice.
