# Supplementary material for: A mouse model of cone photoreceptor function loss (cpfl9) with degeneration due to a mutation in Gucy2e
Source: Front Mol Neurosci. 2023 Jan 9;15:1080136. doi: 10.3389/fnmol.2022.1080136 (PMC9868315; doi:10.3389/fnmol.2022.1080136)
Supplement: Supplementary file 3 [file Image_3.PDF]

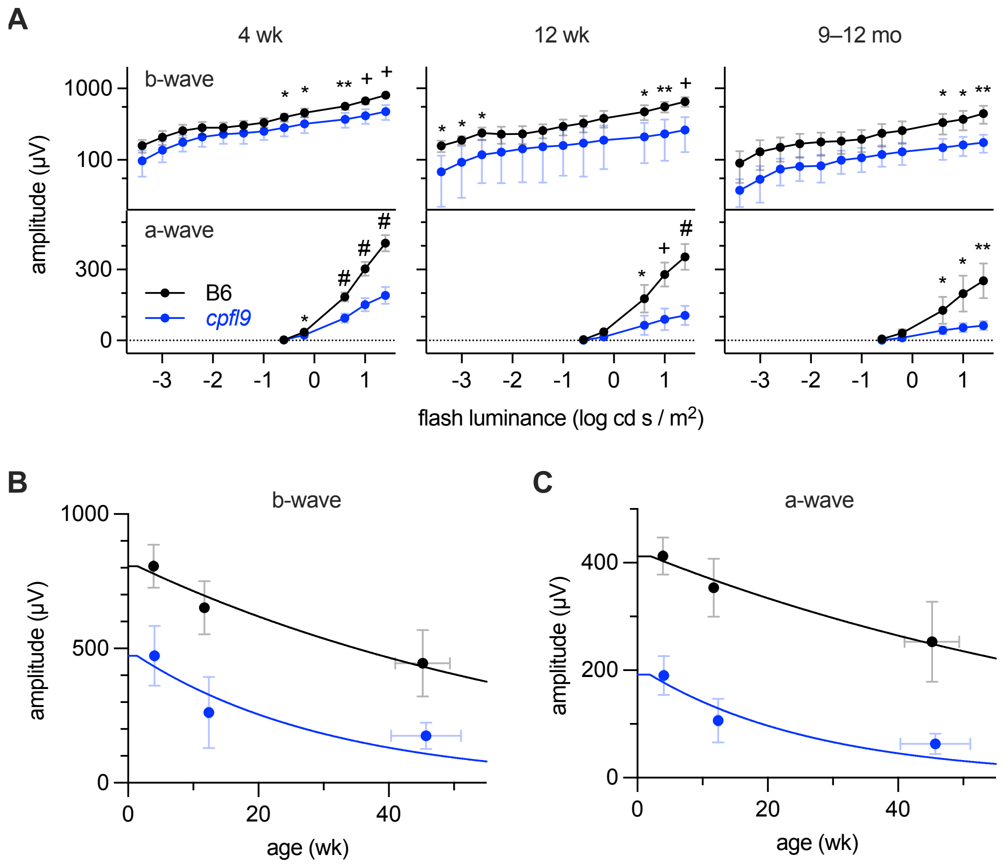

Figure S3. Decrease of scotopic ERG response amplitudes with age in B6 and *cpfl9* mice. (**A**) Plots of scotopic b- and a-wave response amplitudes as a function of flash intensity at 4 weeks (wk; data from Figure 1 is included here for comparison), 12 weeks, and 9–12 months (mo) of age. Values indicate mean ± SD. The responses of both strains decreased with age. Two-way repeated-measures ANOVA indicated a statistically significant effect of strain on each response at 12 weeks (scotopic b-wave, F(1, 12) = 13.9, *p* = 0.0029; scotopic a-wave, F(1, 12) = 47.3, *p* < 0.0001, n = 6 *cpfl9* and n = 8 B6), and 9–12 months (scotopic b-wave, F(1, 10) = 11.6, *p* = 0.0.0066; scotopic a-wave, F(1, 10) = 19.4, *p* = 0.0013; n = 5 *cpfl9* and n = 7 B6). Post hoc multiple-comparison testing (Šídák) revealed statistically significant differences in response amplitude between strains as indicated by symbols (*, *p* < 0.05; **, *p* < 0.01; +, *p* < 0.001; #, *p* < 0.0001). To test for differences in the rate of ERG decline, the scotopic response amplitude at the maximum flash intensity was plotted as a function of age for the b-wave (**B**) and a-wave (**C**). Symbols and lines are colored as in **A**. Individual data points from all mice were fit to a mono-exponential decay with an initial lag. The response amplitude during the lag was constrained to equal the mean response at 4 weeks of age, and at infinite age, zero. Values indicate mean ± SD on both axes. Mono-exponential rate constants (*k*) for the decay in amplitude with age were higher in *cpfl9* mice than in B6 controls, but a statistically significant difference was observed only for the a-wave (b-wave: B6, *k* = 0.014 ± 0.003 wk^{-1} [mean ± SEM], *cpfl9*, *k* = 0.033 ± 0.011 wk^{-1}, *p* = 0.11, Welch's t-test; a-wave: B6, *k* = 0.012 ± 0.002 wk^{-1}, *cpfl9*, *k* = 0.038 ± 0.010 wk^{-1}, *p* = 0.020). As the a-wave reflects rod photoreceptor cell function, these results indicate a more rapid degeneration of rod cells in *cpfl9* compared to B6 mice.
