# Supplementary material for: A mouse model of cone photoreceptor function loss (cpfl9) with degeneration due to a mutation in Gucy2e
Source: Front Mol Neurosci. 2023 Jan 9;15:1080136. doi: 10.3389/fnmol.2022.1080136 (PMC9868315; doi:10.3389/fnmol.2022.1080136)
Supplement: Supplementary file 4 [file Image_4.PDF]

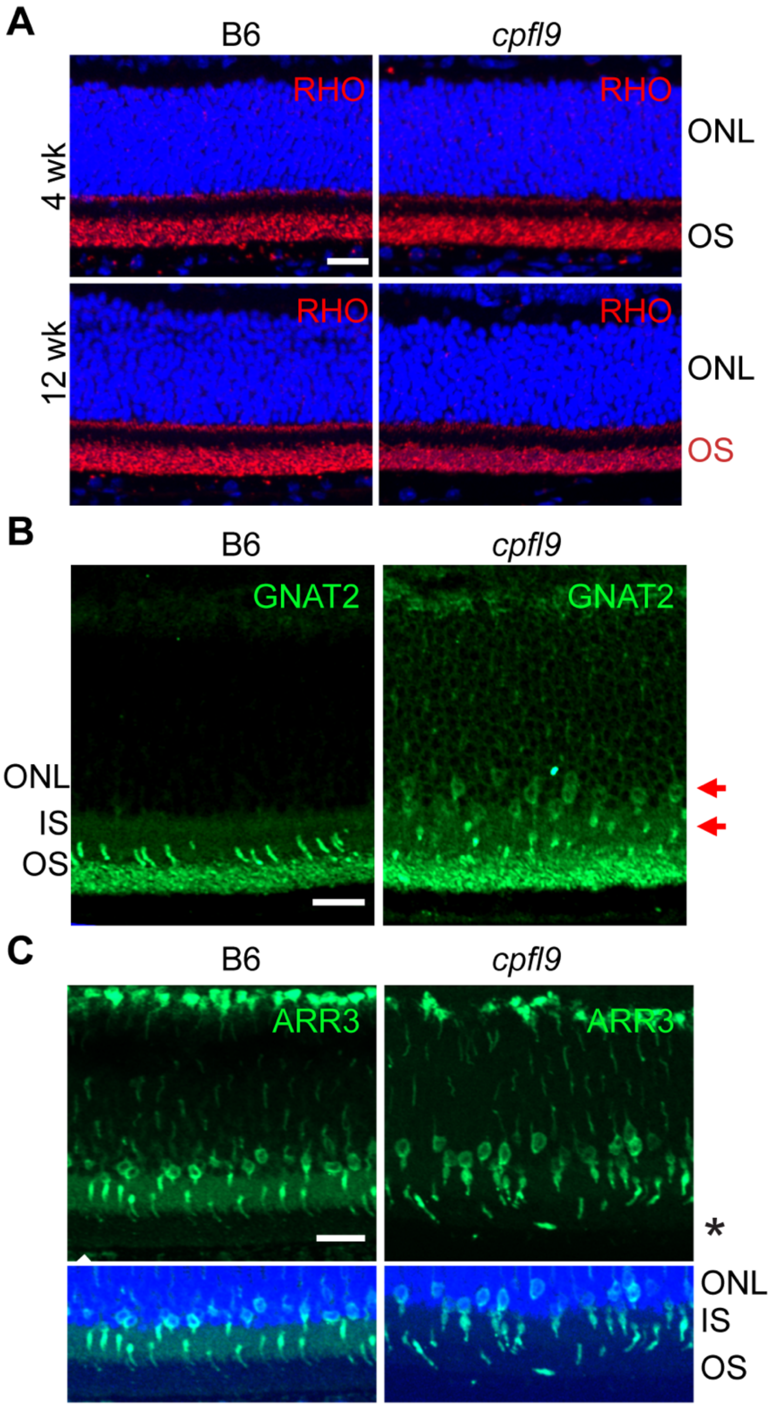

Figure S4. Localization of opsins and OS proteins. Immunostaining retinas of *cpfl9* and B6 control mice with (**A**) anti-rhodopsin (RHO, red), (**B**) anti-G protein subunit alpha transducin 2 (GNAT2, green), (**C**) anti-cone arrestin (ARR3, green) at 12 weeks of age. (**C**-lower panel; anti-ARR3 (green) staining is shown with DAPI (blue) to mark the cone ONL boundaries). Red arrows show mislocalization of GNAT2 in the inner segment and cone cell bodies in the ONL. Asterisk demarcates ARR3 positive staining in distal OS at the RPE interface. Images are representative of n = 4 *cpfl9* and n = 6 B6 mice. Scale bars: 20 μM.
