# Supplementary material for: A mouse model of cone photoreceptor function loss (cpfl9) with degeneration due to a mutation in Gucy2e
Source: Front Mol Neurosci. 2023 Jan 9;15:1080136. doi: 10.3389/fnmol.2022.1080136 (PMC9868315; doi:10.3389/fnmol.2022.1080136)
Supplement: Supplementary file 5 [file Image_5.PDF]

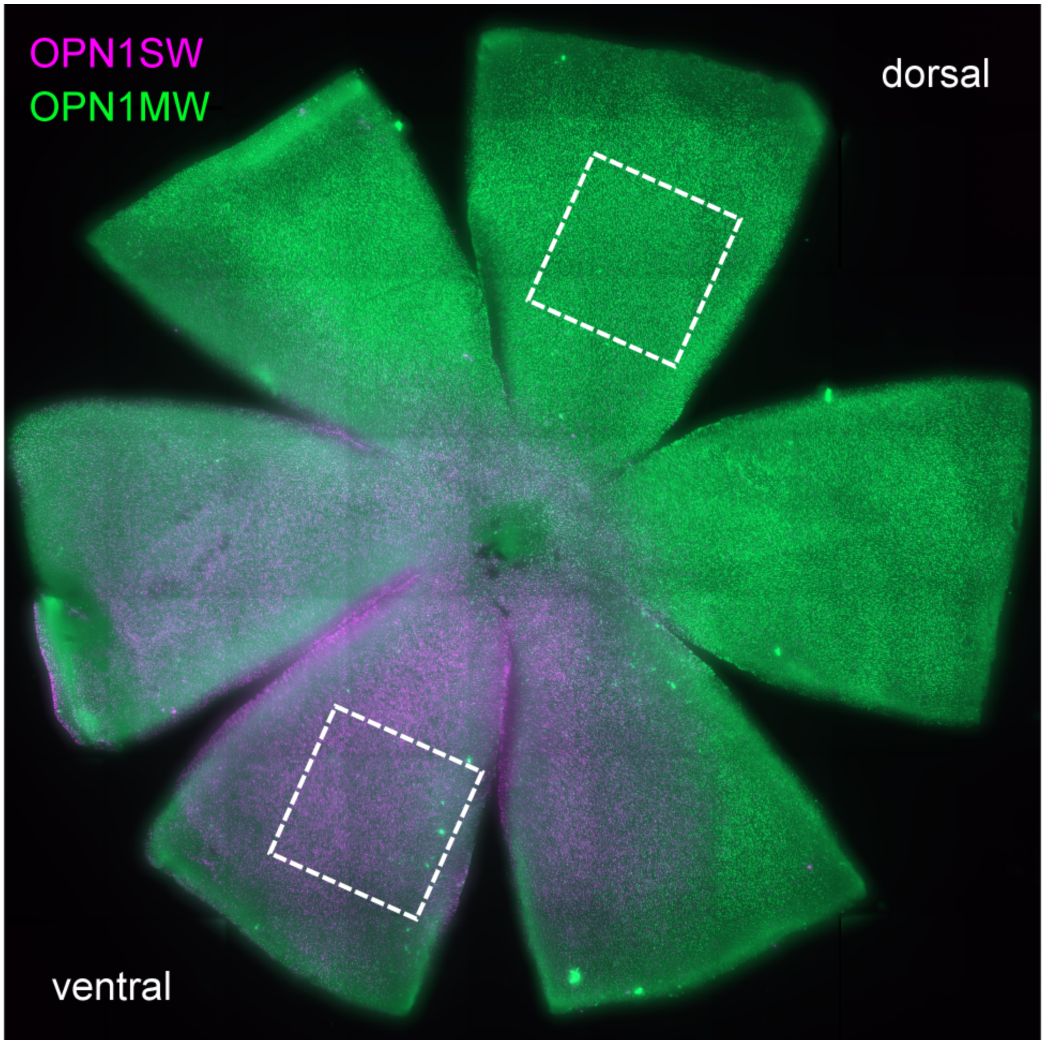

Figure S5. Representative retinal flatmount of a B6 control at 12 weeks of age stained with anti-OPN1MW (green) and anti-OPN1SW (magenta). Boxed region shows approximate locations of dorsal and ventral regions used for imaging cone opsins and determining counts of cone matrix sheaths stained with peanut agglutinin (PNA).
